# Deep convolutional neural network-based automated identification and classification of mungbean foliar diseases

**DOI:** 10.3389/frai.2026.1848787

**Published:** 2026-09-03

**Authors:** Shail Bala, S. I. Harlapur, Aditya Kamalakar Kanade, M. P. Potdar, Gurupada B. Balol, V. B. Kuligod, Lingareddy Usha Rani

**Affiliations:** 1 Department of Plant Pathology, University of Agricultural Sciences, Dharwad, Karnataka, India; 2 Department of Agronomy, Mahatma Phule Krishi Vidyapeeth, Rahuri, Maharashtra, India; 3 Department of Agricultural Meteorology, University of Agricultural Sciences, Dharwad, Karnataka, India; 4 Soil Science and Agricultural Chemistry, University of Agricultural Sciences, Dharwad, Karnataka, India; 5 Division of Plant Pathology, ICAR-Indian Agriculture Research Institute, New Delhi, India

**Keywords:** DCNN, deep learning, disease detection, mungbean, precision agriculture

## Abstract

Early and accurate detection of plant diseases is critical in precision agriculture to improve crop management and yield. Mungbean (*Vigna radiata* L.) is highly susceptible to several foliar diseases, including yellow mosaic, powdery mildew, leaf crinkle, and cercospora leaf spot, which cause substantial productivity losses. Despite expanding applications of deep learning in plant disease diagnosis, systematic multi-architecture evaluation for mungbean disease classification under natural field conditions remains limited. This study addresses this gap by evaluating five state-of-the-art deep convolutional neural network (DCNN) architectures on a large-scale, field-acquired mungbean dataset that captures real-world variability across environmental conditions and disease severity levels, distinguishing it from controlled laboratory studies. A total of 5,617 original images across five classes were used. Data augmentation was applied exclusively to the training subset after stratified splitting to prevent data leakage. The dataset was partitioned into training (70%), validation (15%), and testing (15%) subsets. VGG16, VGG19, ResNet50V2, DenseNet121, and InceptionV3 were evaluated using identical transfer learning and fine-tuning protocols. Model performance was assessed using AUC-ROC, Cohen's kappa coefficient, McNemar's test for pairwise statistical comparisons, five-fold cross-validation, and Grad-CAM-based interpretability. On the independent test set, InceptionV3 achieved the highest accuracy (98.47%) and macro-F1 (98.49%), followed by VGG16 (98.36%) and VGG19 (97.89%). AUC-ROC values exceeded 0.997 for all models, confirming excellent class discrimination. Grad-CAM visualizations further confirmed that model predictions were based on biologically relevant disease symptoms. The findings demonstrate the effectiveness of deep learning for robust disease recognition under realistic field conditions and highlight the potential of AI-based diagnostic tools for crop health monitoring, precision agriculture, and decision-support systems in mungbean production.

## 1 Introduction

Ensuring global food security has become an increasingly critical challenge in the twenty-first century. The world's population is expected to exceed 9.7 billion by the middle of this century, creating unprecedented pressure on agricultural systems to supply sufficient food, feed, and fiber (Dorling, 2021). Global food production must rise by approximately 70% by 2050 to meet nutritional demand, underscoring the urgent need for sustainable agricultural intensification (McKenzie and Williams, 2015; Kopittke et al., 2019). Among pulse crops, mungbean (*Vigna radiata* L.), popularly known as greengram, is an important pulse crop due to its short growth duration, rich protein content, nitrogen-fixing ability, and adaptability to diverse agro-climatic conditions (Huppertz et al., 2023). However, its productivity is severely constrained by several foliar diseases, including Mungbean Yellow Mosaic Disease (MYMD), powdery mildew, leaf crinkle, and cercospora leaf spot, which collectively cause substantial yield losses and affect crop quality (Chauhan et al., 2025). In severe epidemics, MYMD alone can cause 60%−80% yield loss (Dubey and Singh, 2013), while combined foliar infections may reduce productivity by more than 50%, indicating a significant economic burden for mungbean-growing regions (Pandey et al., 2018).

Conventional disease diagnosis in mungbean depends on visual field inspection by trained specialists, which is labor-intensive, subjective, and prone to human error. More critically, visual assessment can only detect diseases once symptoms have already manifested, potentially delaying timely intervention. Image-based deep learning methods offer a more consistent and objective means of disease identification once visible symptoms appear (Kanade et al., 2025). Although chemical control remains widely used, excessive and untimely application leads to environmental contamination, pesticide resistance, and increased production costs (Devi et al., 2022). There is therefore a pressing need for precise, automated, and scalable disease detection systems to enable timely decision-making and support sustainable crop management (Vinod et al., 2024).

Artificial intelligence (AI) and machine learning (ML) have emerged as transformative technologies for smart agriculture in the era of the fourth agricultural revolution (Kamilaris and Prenafeta-Boldú, 2018; Liakos et al., 2018). Deep Learning (DL), a subset of AI that mimics the hierarchical learning of the human brain, has shown remarkable success in agricultural applications such as crop and weed identification, pest and disease detection, and yield estimation (Ferentinos, 2018; Too et al., 2019). Unlike traditional image processing approaches that require manual feature extraction–DL models automatically learn discriminative visual features from large datasets, offering robust performance under complex field conditions. Among DL techniques, Deep Convolutional Neural Networks (DCNNs) have become the most powerful and widely used framework for image-based plant disease detection (Tugrul et al., 2022). Architectures such as VGG16, ResNet, DenseNet, and Inception have demonstrated exceptional ability in learning complex visual representations from leaf images, even under challenging conditions of variable lighting, background clutter, and disease overlap (Brahimi et al., 2018; Mohanty et al., 2016).

Traditional machine learning methods such as Support Vector Machines (SVM), Random Forest (RF), and k-Nearest Neighbors (kNN) depend heavily on handcrafted features like color, shape, and texture (Arnal Barbedo, 2013; Rumpf et al., 2010). In contrast, DCNNs automatically extract hierarchical features— edges, textures, and patterns— from raw pixels, making them more efficient for detecting subtle disease symptoms across different genotypes and growth stages (Deb and Rahman, 2025). Each major architecture embodies distinct design philosophies: VGG models (Simonyan and Zisserman, 2015) employ uniform stacks of small convolutional filters for reliable baseline performance; ResNet (He et al., 2016) uses skip connections to address gradient degradation in deep networks; DenseNet (G. Huang et al., 2017; X. Huang et al., 2023) connects every layer to all subsequent layers, promoting feature reuse; and InceptionV3 (Szegedy et al., 2016) applies parallel multi-scale convolutions, enabling simultaneous capture of fine lesion textures and broad leaf-scale patterns.

Numerous studies have demonstrated the effectiveness of DCNN-based approaches for disease detection across a wide range of crops, including tomato, potato, rice, wheat, and several others (Dolatabadian et al., 2025). Deep learning-based disease classification has also been explored in mungbean (Mallick et al., 2025). However, comprehensive evaluation of multiple state-of-the-art DCNN architectures using a large field-acquired mungbean dataset under natural field conditions remain limited. In addition, relatively few studies have performed statistically validated comparisons among multiple architectures or incorporated comprehensive evaluation criteria beyond classification accuracy. With the increasing availability of imaging technologies, deep learning-based approaches have considerable potential for field-level disease monitoring and precision agriculture (Upadhyay et al., 2025). Furthermore, evaluation metrics such as AUC–ROC, Cohen's kappa coefficient, model complexity analysis, and statistical significance testing provide a more rigorous assessment of model performance and practical utility (Kheir et al., 2025).

The present study addresses these limitations by employing a large-scale, field-representative image dataset capturing variation across multiple mungbean genotypes, diverse environmental backgrounds, and a broad spectrum of symptom intensities. Five state-of-the-art pre-trained DCNN architectures (VGG16, VGG19, ResNet50V2, DenseNet121, and InceptionV3) were evaluated under a rigorous and uniform protocol for automated classification of four major foliar diseases in mungbean. Rather than proposing a new architecture, the study fine-tunes existing pre-trained models to assess their transferability to field-based mungbean disease images, contributing to reproducible benchmarking for this crop. Evaluation was extended beyond conventional accuracy metrics to include AUC-ROC, Cohen's kappa, per-class F1-score analysis, model complexity metrics, and McNemar's pairwise statistical significance tests. The specific objectives were: (i) to develop a robust and reproducible deep learning pipeline for multi-class mungbean foliar disease classification; (ii) to systematically compare the performance of five DCNN architectures under identical experimental conditions; and (iii) to validate performance differences using formal statistical significance testing.

The remainder of this paper is organized as follows: Section 2 describes the materials and methods, including dataset preparation, pre-processing, augmentation strategy, architecture selection rationale, and training protocol. Section 3 presents results encompassing overall and class-wise model performance, confusion matrix analysis, ROC curves, and statistical tests. Section 4 discusses comparative model performance and its implications. Section 5 concludes with key findings and future research directions.

## 2 Materials and methods

### 2.1 Dataset collection and description

A comprehensive dataset of mungbean (*Vigna radiata* L.) leaf images was developed to support deep learning-based classification of major foliar diseases. Images of mungbean yellow mosaic and leaf crinkle were captured during the summer season (March–June), powdery mildew during the late *Kharif* season (September–October), and cercospora leaf spot during the *Kharif* season (June–October) of the 2024–25. Healthy leaf images were collected across multiple crop growth stages in different seasons. All images were captured from the experimental fields of the University of Agricultural Sciences, Dharwad. The study site, located in the Northern Transitional Zone of Karnataka at 15° 30' 6” N latitude and 74° 59' 13.2” E longitude, an agro-climatic zone that provides suitable conditions for mungbean cultivation and natural disease incidence.

**Figure 1:**
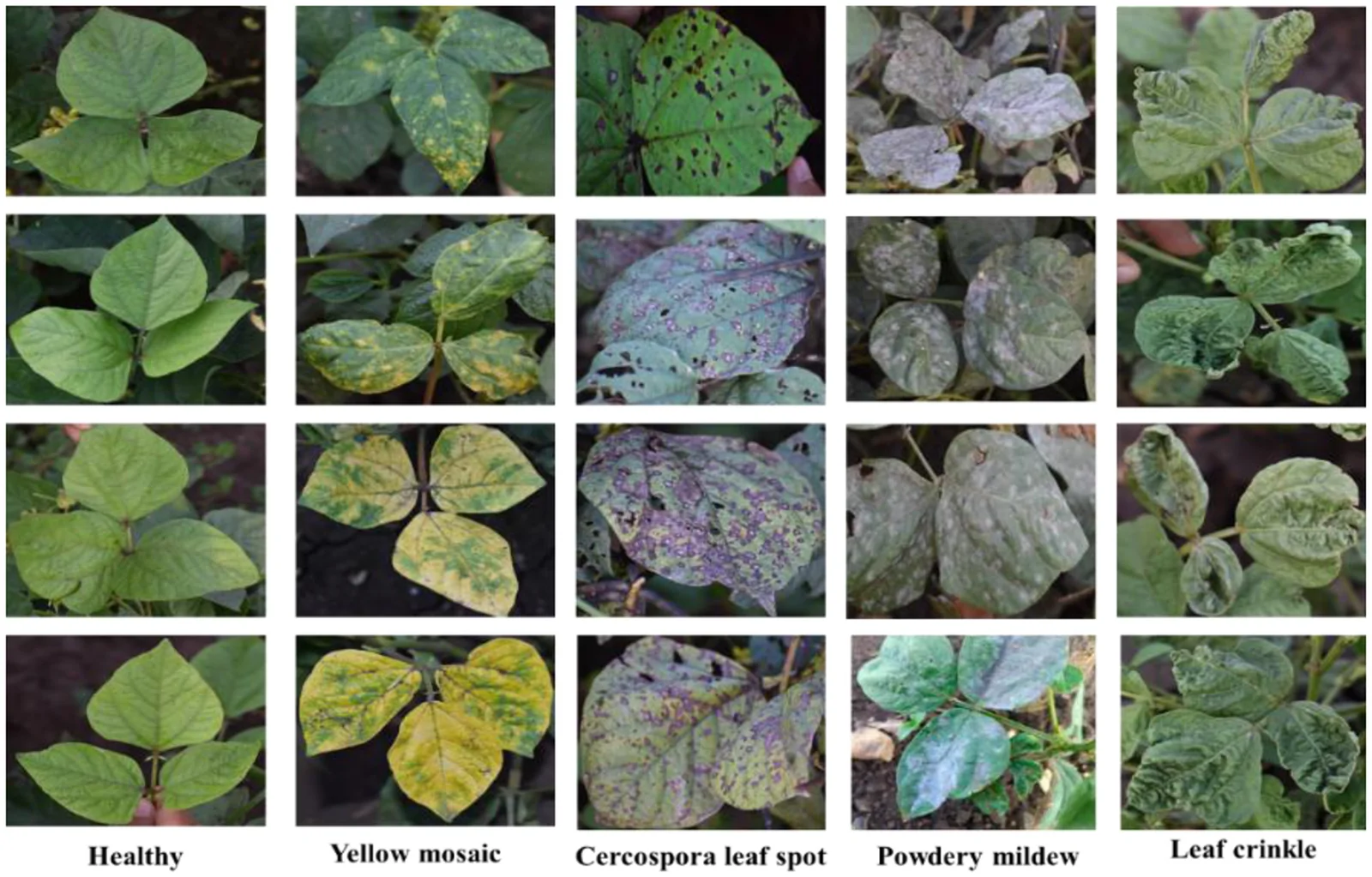
Representative images from the dataset showing variability in disease symptoms and imaging conditions across classes.

Disease identification and labeling were carried out based on characteristic visual symptoms by trained plant pathologists, following standard diagnostic criteria described by Kamireddy et al. (2025) and Parasappa Saable et al. (2024). Field sampling was carried out across diverse genotypes and growth stages to ensure a broad representation of symptoms and natural variations. High-resolution images were captured using a Nikon D7500 DSLR camera equipped with an 18–55 mm focal length (Table 1). Images were captured under variable field lighting conditions, between 9:00 a.m. and 4:00 p.m., ensuring inclusion of both shaded and direct sunlight images to improve the dataset's robustness. Each image was captured from approximately 30–40 cm above the leaf surface to maintain consistent scale and focal distance. In total, 5,617 original images were collected, encompassing healthy leaves and leaves infected with the four target diseases, including mungbean yellow mosaic, leaf crinkle, powdery mildew, and cercospora leaf spot (Figure 1 and Table 2).

**Table 1:** Camera parameters for image acquisition.

Specification	Parameter
Camera model	Nikon D7500
F-stop	f/5.6
Exposure time	1/4,000 s
ISO range	100–25,600
Focal length	18–55 mm
Max aperture	3.5
Contrast	Normal
Saturation	Normal
White balance	Auto

**Table 2:** Total number of original images of the mungbean dataset.

Images categories	No. of original images
Healthy	1,206
Yellow mosaic	1,659
Leaf crinkle	437
Powdery mildew	1,460
Cercospora leaf spot	855

### 2.2 Image pre-processing and augmentation, and dataset splitting

To prepare the dataset for model training, several pre-processing steps were performed. All images were resized to 256 × 256 pixels to maintain uniform input dimensions across models and to balance computational efficiency with feature clarity. Image pre-processing and augmentation were implemented using the Keras ImageDataGenerator, following the approach described by Shorten and Khoshgoftaar (2019).

**Figure 2:**
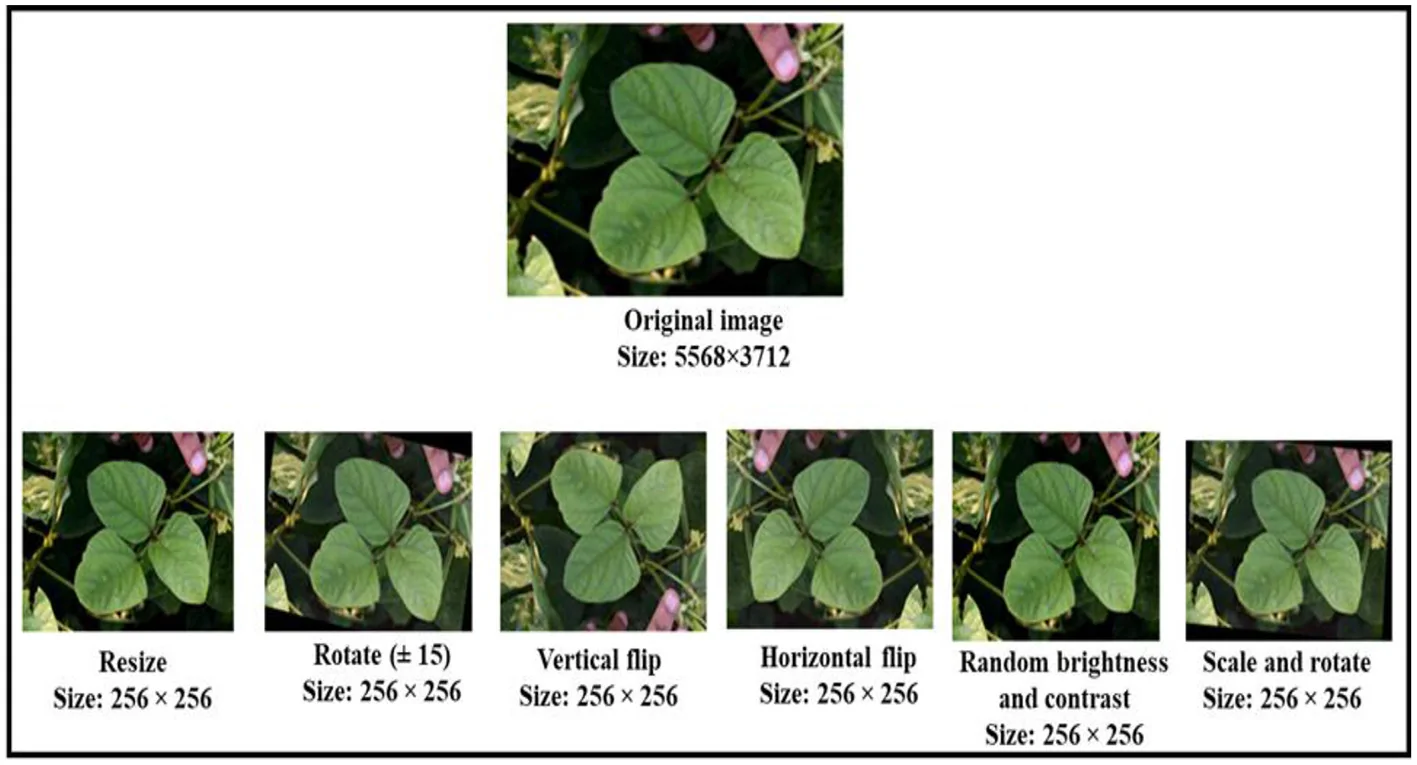
Pre-processing steps for dataset augmentation and overfitting mitigation.

To improve model generalizability and increase training sample diversity, data augmentation was applied exclusively to the training subset after stratified splitting. Original, unaugmented images were first partitioned into training (70%), validation (15%), and test (15%) subsets using stratified random sampling to ensure proportional class representation across all subsets (Table 3). Augmentation was subsequently performed only on the training partition to increase sample diversity and enhance model robustness. Augmentation operations applied to the training subset included random rotations (±15°), horizontal and vertical flips, brightness and contrast adjustments, and scale-and-rotate transformations (Figure 2). These operations simulated natural variations in leaf orientation, field illumination, and imaging perspective, thereby improving model robustness under real-world conditions. Because augmentation was applied uniformly across all classes, the relative class distribution remained unchanged. The validation and test subsets contained only original, unaugmented images, ensuring an unbiased assessment of model performance.

**Table 3:** Distribution of original images per class across training, validation, and test subsets.

Classes	Training	Validation	Testing
Number of Images			
Healthy	844	181	181
Yellow mosaic	1,161	248	250
Cercospora leaf spot	598	128	129
Powdery mildew	1,022	219	219
Leaf crinkle	305	65	67

No explicit oversampling or undersampling techniques were employed. Instead, class imbalance was mitigated through the use of class-weighted learning during model training, while model performance was evaluated using class-wise precision, recall, and F1-score metrics in addition to overall accuracy.

### 2.3 DCNN architectures and implementation details

Five pre-trained DCNN architectures—VGG16, VGG19, ResNet50V2, DenseNet121, and InceptionV3 were evaluated for performance (Table 4). Architecture selection was guided by four criteria: (i) demonstrated effectiveness in prior plant disease classification studies; (ii) diversity of architectural design principles (depth, skip connections, dense connectivity, multi-scale processing); (iii) suitability for the available computational hardware; and (iv) compatibility with Keras/TensorFlow for standardized implementation. VGG16 was selected for its simplicity, strong baseline performance in plant-disease literature, and suitability for Grad-CAM visualization due to large spatial feature maps. VGG19 was included as a deeper VGG variant offering additional convolutional depth for capturing finer textural features. ResNet50V2 was chosen for its efficient residual learning with pre-activation blocks and superior performance relative to VGG counterparts in published benchmarks. DenseNet121 was selected for its dense connectivity pattern that maximizes feature reuse while requiring the fewest parameters among the evaluated architectures, making it particularly suitable for deployment on resource-constrained devices. InceptionV3 was included for its parallel multi-scale Inception modules capable of simultaneously capturing fine lesion textures and broad leaf-scale patterns.

**Table 4:** Evaluated DCNN architectures and corresponding Keras reference URLs.

Sl. No.	Model	URL
1	VGG16	https://keras.io/api/applications/vgg/#vgg16-function
2	VGG19	https://keras.io/api/applications/vgg/#vgg19-function
3	ResNet50V2	https://keras.io/api/applications/resnet/#resnet50v2-function
4	InceptionV3	https://keras.io/api/applications/inceptionv3/
5	DenseNet121	https://keras.io/api/applications/densenet/

#### 2.3.1 VGG16

The VGG16 model proposed by Simonyan and Zisserman (2015) is a 16-layer deep CNN comprising 13 convolutional and three fully connected layers. It accepts a fixed-size RGB image as input and employs a uniform 3 × 3 convolutional kernel throughout the architecture, which helps in capturing fine spatial details while maintaining computational efficiency. Each convolution operation is performed with a stride of one pixel, and padding of one pixel is applied to preserve spatial dimensions across layers.

**Figure 3:**
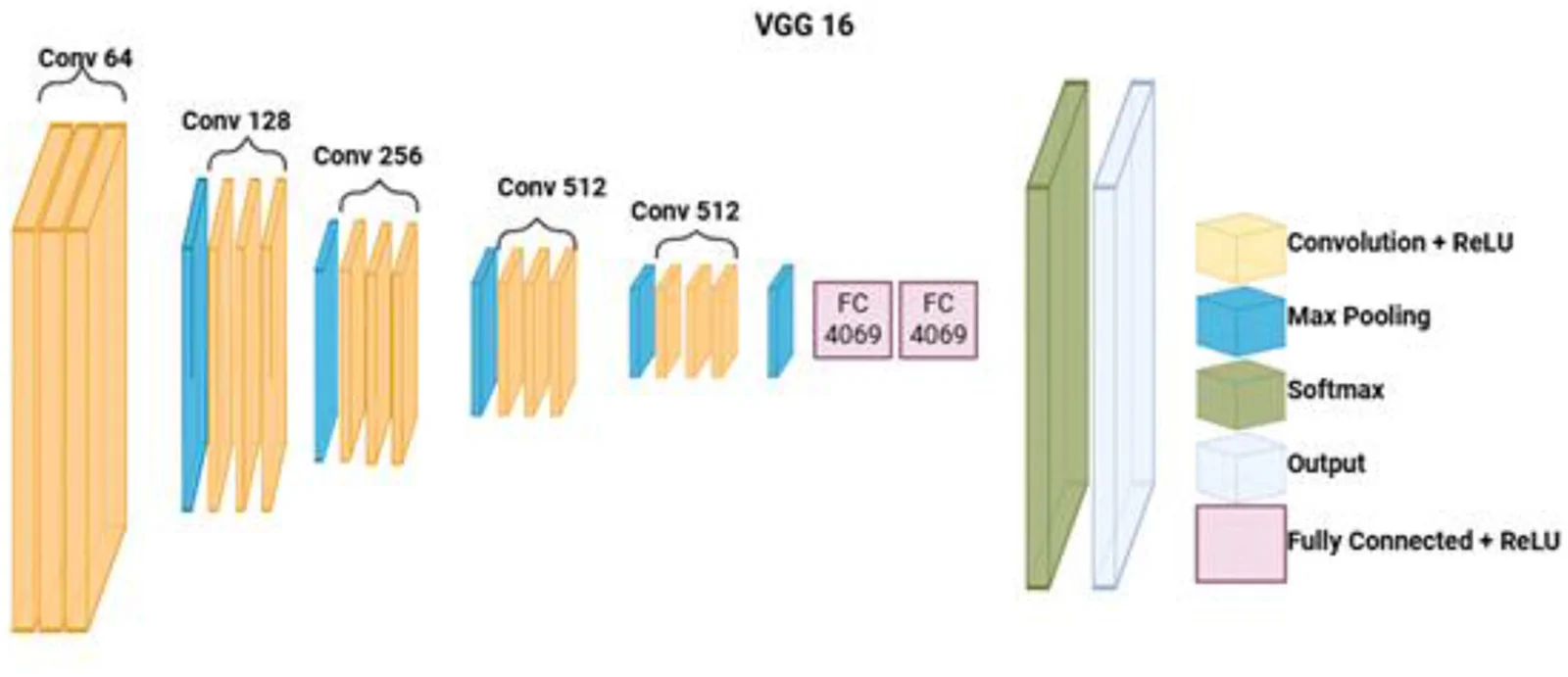
Network structure of VGG 16 DCNN model.

The network begins with two convolutional layers, each containing 64 filters, followed by a 2 × 2 max pooling layer with a stride of two for spatial down-sampling. This pattern is extended in subsequent blocks, where two convolutional layers with 128 filters are followed by pooling, and then three convolutional layers with 256 filters are added. The deeper layers of the model consist of two sets of three convolutional layers, each comprising 512 filters of size 3 × 3, separated by max pooling layers of the same configuration. After the convolutional feature extraction stage, the network transitions to the fully connected (FC) layers, consisting of two layers with 4,069 neurons each, followed by a final FC layer responsible for classification. The output layer employs a Softmax activation to produce probability distributions over the target classes. All hidden layers use the Rectified Linear Unit (ReLU) activation function to introduce non-linearity and accelerate training convergence (Figure 3). In the present study, the convolutional base was initially frozen during Phase 1 training and partially unfrozen during Phase 2 fine-tuning, and the classification head was replaced for 5-class output.

#### 2.3.2 VGG19

**Figure 4:**
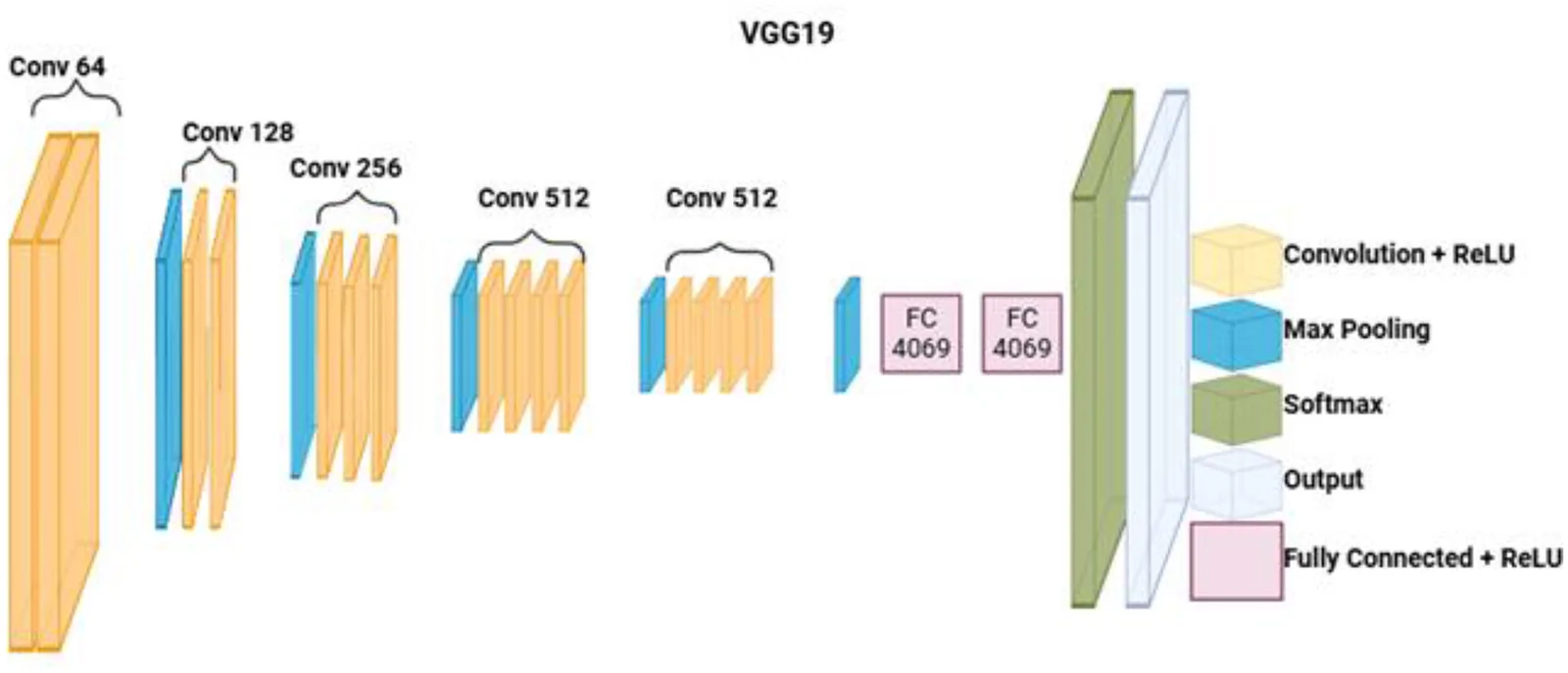
Network structure of VGG 19 DCNN model.

The VGG19 model, an extended version of VGG16 proposed by Simonyan and Zisserman (2015), is a deeper convolutional neural network comprising 19 learnable layers (16 convolutional and three fully connected layers). It follows the same design philosophy as VGG16, employing small 3 × 3 convolutional kernels throughout the network with a stride of one and uniform padding to preserve spatial dimensions. The use of smaller filters stacked in multiple layers enables the network to effectively capture complex hierarchical features while keeping computational costs relatively manageable compared to larger kernel designs. The architecture begins with two convolutional layers containing 64 filters, followed by a 2 × 2 max pooling layer for spatial reduction. This is succeeded by two convolutional layers with 128 filters, three layers with 256 filters, and four convolutional layers each with 512 filters in the subsequent two blocks. Every convolutional layer is followed by a Rectified Linear Unit (ReLU) activation function, which introduces non-linearity and speeds up convergence. Max pooling is applied after each convolutional block to progressively reduce feature map size while retaining essential information. After the convolutional feature extraction stages, the model includes three fully connected (FC) layers: two with 4,069 neurons each and a final classification layer with Softmax activation for multi-class output (Figure 4). Compared to VGG16, the additional layers in VGG19 enhance its capacity to learn deeper and more abstract representations, improving performance on complex visual tasks. However, this comes at the cost of increased computational demand and training time due to the higher number of parameters. Parameter count increases to approximately 20.4 M, at higher computational cost, but the model benefits from strong ImageNet pre-training and remains compatible with Grad-CAM visualization.

#### 2.3.3 ResNet50V2

**Figure 5:**
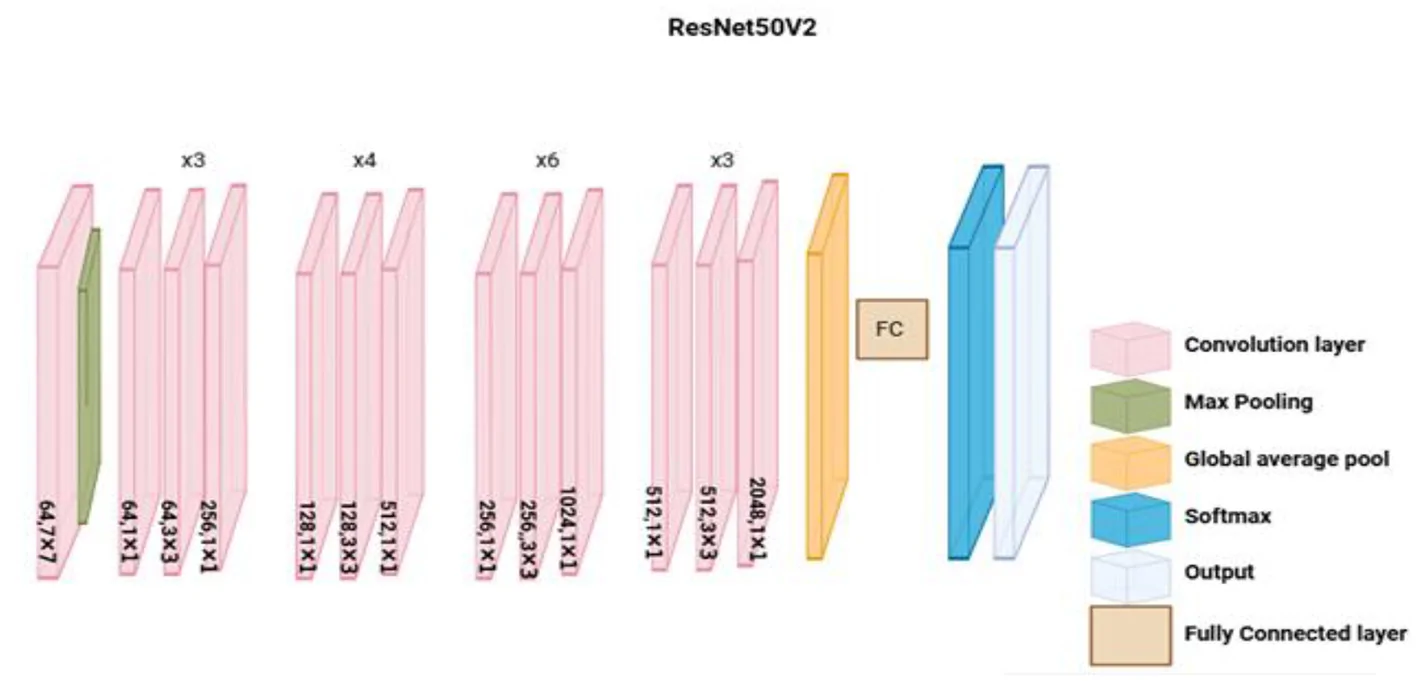
Network structure of ResNet50V2 DCNN model.

The ResNet50V2 architecture, introduced by He et al. (2016), represents a major advancement in deep learning through the use of residual learning. Unlike traditional convolutional neural networks, ResNet incorporates skip (shortcut) connections that allow the network to bypass one or more layers, effectively addressing the vanishing gradient problem and enabling the successful training of very deep networks. ResNet50V2 is composed of 50 layers, including convolutional, batch normalization, and identity mapping blocks. The model begins with a 7 × 7 convolutional layer and a 3 × 3 max pooling layer for initial feature extraction. The main body of the architecture consists of bottleneck residual blocks, each containing 1 × 1, 3 × 3, and 1 × 1 convolutions. The 1 × 1 layers serve to reduce and then restore dimensionality, while the 3 × 3 convolution focuses on feature transformation (Figure 5). In ResNetV2, the order of operations is slightly modified from the original ResNet; batch normalization and ReLU activation are applied before each convolution, leading to improved gradient flow and stability during training. The final stage includes global average pooling followed by a fully connected layer and a Softmax classifier. With approximately 24.7 M total parameters but only 9.1 M trainable in the fine-tuning configuration used here, it offers efficient learning.

#### 2.3.4 InceptionV3

The InceptionV3 architecture, developed by Szegedy et al. (2016), builds on the principles of the original Inception network by introducing several key optimizations to improve efficiency and representational power. It employs a multi-branch convolutional structure that processes the input at multiple spatial scales simultaneously, enabling the model to capture both coarse- and fine-scale features within an image.

**Figure 6:**
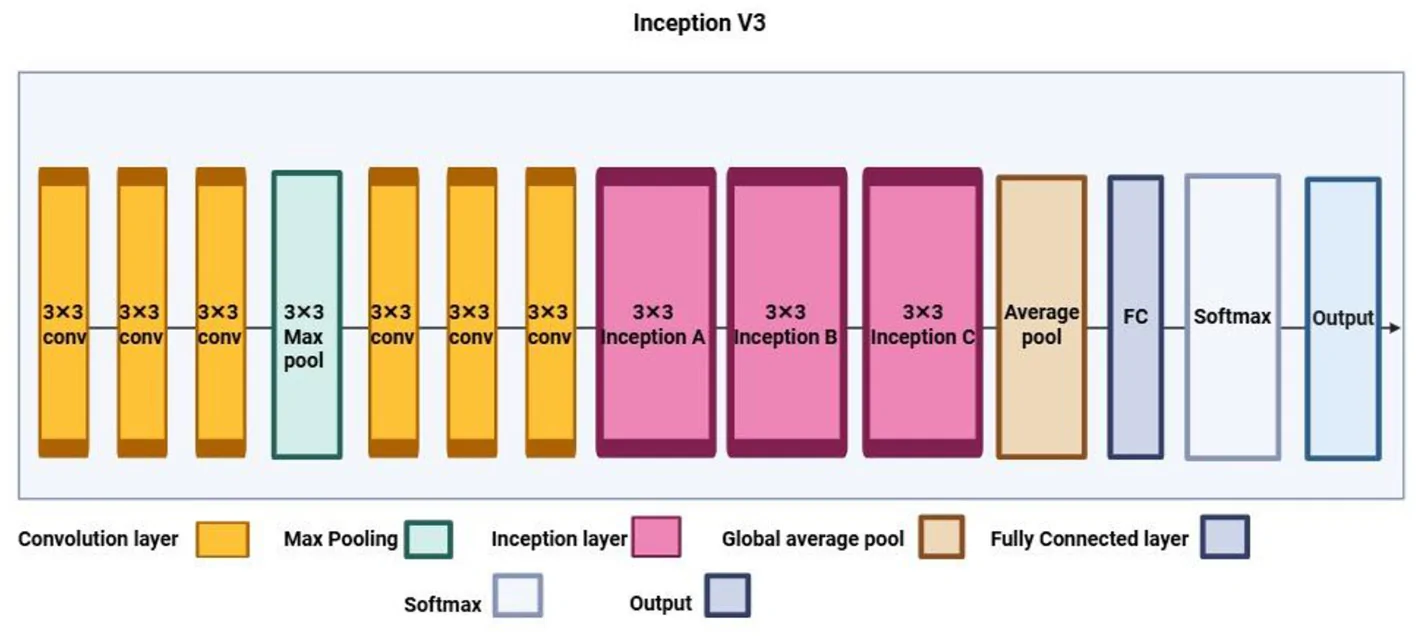
Network structure of InceptionV3 DCNN model.

A hallmark of InceptionV3 is its use of factorized convolutions, where larger kernels (e.g., 5 × 5) are decomposed into smaller ones (e.g., 3 × 3 or 1 × 3 and 3 × 1). This reduces computational cost while retaining the same receptive field. The model also incorporates batch normalization, label smoothing, and auxiliary classifiers to enhance regularization and prevent overfitting. The architecture consists of a series of Inception modules, each combining different convolutional operations in parallel and concatenating their outputs along the channel dimension. The network concludes with a global average pooling layer, a fully connected layer, and a Softmax classifier for final predictions (Figure 6).

By efficiently balancing depth, width, and resolution, InceptionV3 achieves high accuracy with reduced computational demand. Its ability to analyze multi-scale features makes it particularly effective for distinguishing visually similar disease symptoms in complex agricultural imagery.

#### 2.3.5 DenseNet121

**Figure 7:**
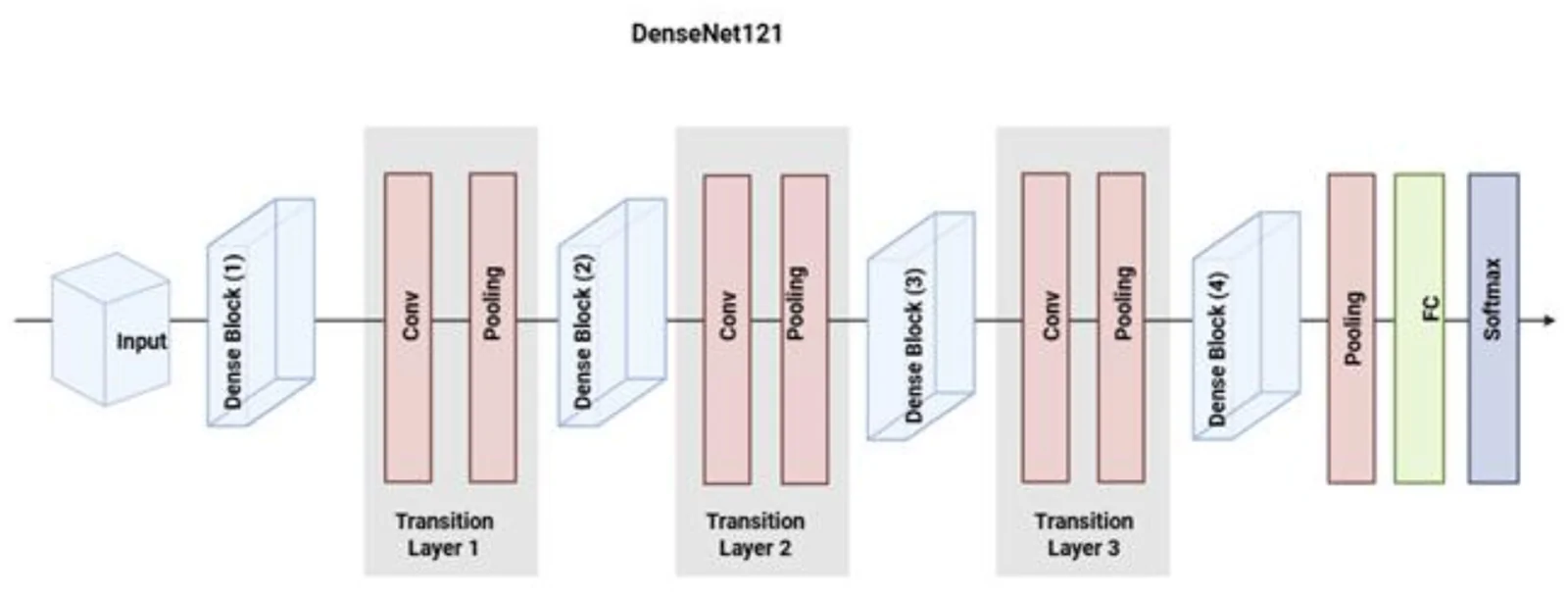
Network structure of DenseNet121 DCNN model.

The DenseNet121 model proposed by Huang et al. (2017) is characterized by dense connectivity between layers, where each layer receives input from all preceding layers and passes its output to all subsequent ones. This innovative design improves feature reuse, gradient propagation, and parameter efficiency, leading to better learning and generalization with fewer parameters compared to traditional CNNs. DenseNet121 comprises four dense blocks, each consisting of multiple convolutional layers interlinked through direct connections. Within each dense block, feature maps from previous layers are concatenated rather than summed, which promotes the preservation of both low-level and high-level features throughout the network. Between dense blocks, transition layers comprising 1 × 1 convolution and 2 × 2 average pooling, are used to control model complexity and reduce the number of feature maps (Figure 7). The architecture begins with an initial convolution and pooling layer, followed by the dense and transition blocks, and concludes with global average pooling and a fully connected Softmax layer for classification. This dense connectivity pattern reduces redundancy and mitigates vanishing gradients, allowing the model to learn highly discriminative representations even with limited data. DenseNet121's balance between performance and computational efficiency makes it well-suited for agricultural disease classification tasks involving high visual similarity between classes.

### 2.4 Model training and optimization: two-phase transfer learning

Model training was conducted in the Kaggle environment utilizing GPU T4 × 2 with TensorFlow 2.18 and Keras API. It employed a two-phase transfer learning strategy. In Phase 1 (frozen-base training), the pre-trained ImageNet convolutional base was fully frozen and only the newly appended classification head was trained. The classification head consisted of a global average pooling layer, a dense layer (256 neurons, ReLU), a dropout layer (rate = 0.5), and a 5-class Softmax output. Phase 1 was conducted until the validation loss plateaued, with early stopping (patience = 5 epochs) triggering the transition. The number of Phase 1 epochs varied per model: VGG16 (33 epochs), VGG19 (43 epochs), ResNet50V2 (35 epochs), DenseNet121 (39 epochs), and InceptionV3 (29 epochs). In Phase 2 (fine-tuning), the convolutional base was partially unfrozen and all layers were trained jointly at a reduced learning rate to allow domain-specific adaptation of pre-trained weights to mungbean disease features. The models were trained using the Stochastic Gradient Descent (SGD) optimizer with learning rate = 0.001 and categorical cross-entropy as the loss function. The detailed hardware and software configurations used for model training and evaluation are presented in Tables 5–7.

**Table 5:** Hardware environment.

Sl No.	Hardware	Environment
1.	CPU	Intel Core(TM) i3-N305
2.	RAM	8 GB
3.	GPU	GPU T4 × 2 (Kaggle environment)
4.	Storage	1 TB SSD
5.	Operating system	Windows 11

**Table 6:** Software environment.

Sl. No.	Software	Environment
1.	Programming language	Python 3.10
2.	Deep learning framework	TensorFlow 2.18.0/PyTorch 2.6.0 + cu124
3.	Additional libraries	NumPy, Matplotlib, Pandas

**Table 7:** Description of hyper parameters configuration used in this study.

Sl. No.	Hyperparameter	Value/condition
1	Optimizer	Stochastic gradient descent (SGD)
2	Batch size	32
3	Learning rate (phase 1)	0.001
4	Learning rate (phase 2)	Reduced (model-specific)
5	Epochs	50 (combined both phase)
6	Early stopping patience	5 epochs (val. loss)
7	Loss function	Categorical cross-entropy
8	Dropout rate	0.5

### 2.5 Grad-CAM interpretability analysis

To provide visual explainability and validate the biological relevance of the classification decisions, Gradient-weighted Class Activation Mapping (Grad-CAM) was systematically applied across all five evaluated DCNN architectures. Grad-CAM computes the gradients of the predicted class score with respect to the feature map activations of the network's final convolutional layer, producing a localized heatmap of the region most influential to the prediction. Because spatial resolution is governed by network depth and layer shape, the targeting protocol was tailored to the architectural configuration of each network. This multi-model interpretability framework was applied to representative, correctly classified samples from all five disease classes to verify that model attention consistently corresponded to biologically meaningful symptomatic leaf regions rather than background field artifacts, allowing for a direct comparative evaluation of feature localization across different architectural design principles.

### 2.6 Performance evaluation and statistical analysis

Model performance was evaluated on the held-out test set using standard classification metrics derived from the confusion matrix: accuracy, macro-averaged precision, macro-averaged recall, macro-averaged F1-score, and weighted F1-score. Additionally, Cohen's kappa coefficient (κ) was computed to quantify agreement beyond chance, and macro-averaged AUC-ROC was calculated using one-vs.-rest binary decomposition for each class (Powers, 2020). Per-class F1-scores were also reported to identify class-specific challenges.

To assess the statistical significance of observed performance differences, McNemar's test was applied to all pairwise model comparisons using predictions from the shared test set. McNemar's test was evaluated on the same dataset because it accounts for the dependency between paired predictions. To control the family-wise error rate across multiple comparisons, *p*-values were adjusted using the Holm-Bonferroni correction (α = 0.05).

**Figure 8:**
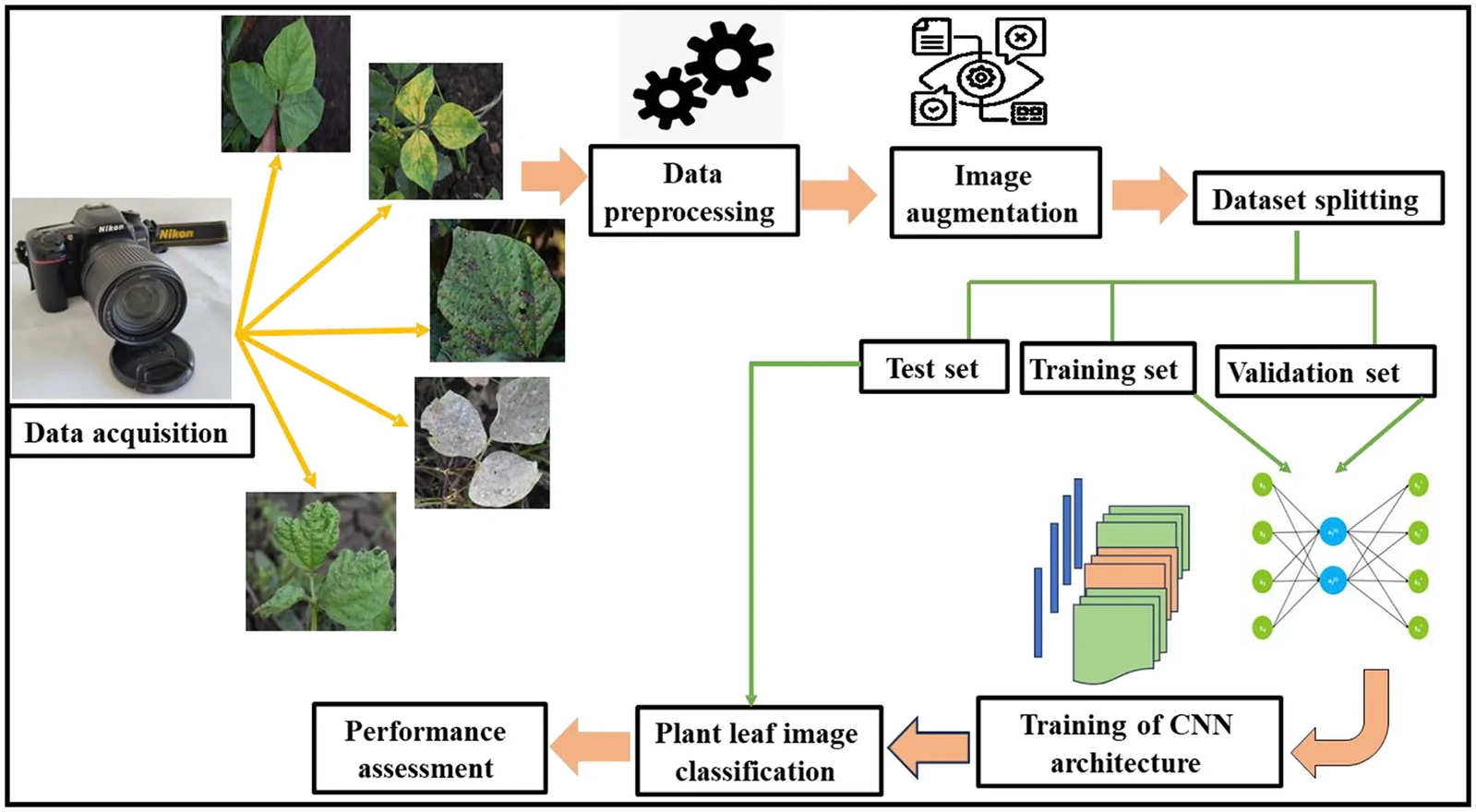
Graphical representation of mungbean foliar disease DCNN classification model.

Additionally, 5-fold stratified cross-validation was performed to assess model stability across training folds. Differences in cross-validation accuracy were evaluated using Friedman's test, followed by Wilcoxon signed-rank *post-hoc* comparisons when appropriate. Model complexity was assessed using total parameter count, model file size (MB), and average per-image inference time (ms). The overall methodology followed in this research is illustrated in the flowchart (Figure 8).

## 3 Results

### 3.1 Training and validation curves

**Figure 9:**
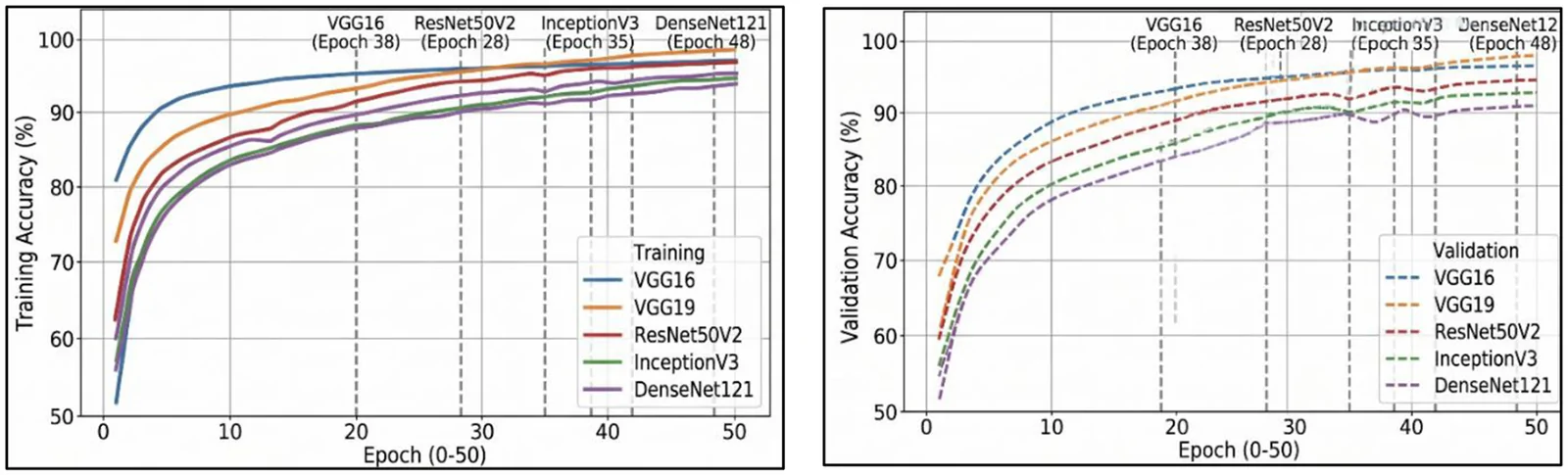
Training and validation accuracy curves across 50 epochs for all five DCNN architectures (VGG16, VGG19, ResNet50V2, DenseNet121, InceptionV3), with vertical dashed lines indicating early stopping epoch for each model.

**Figure 10:**
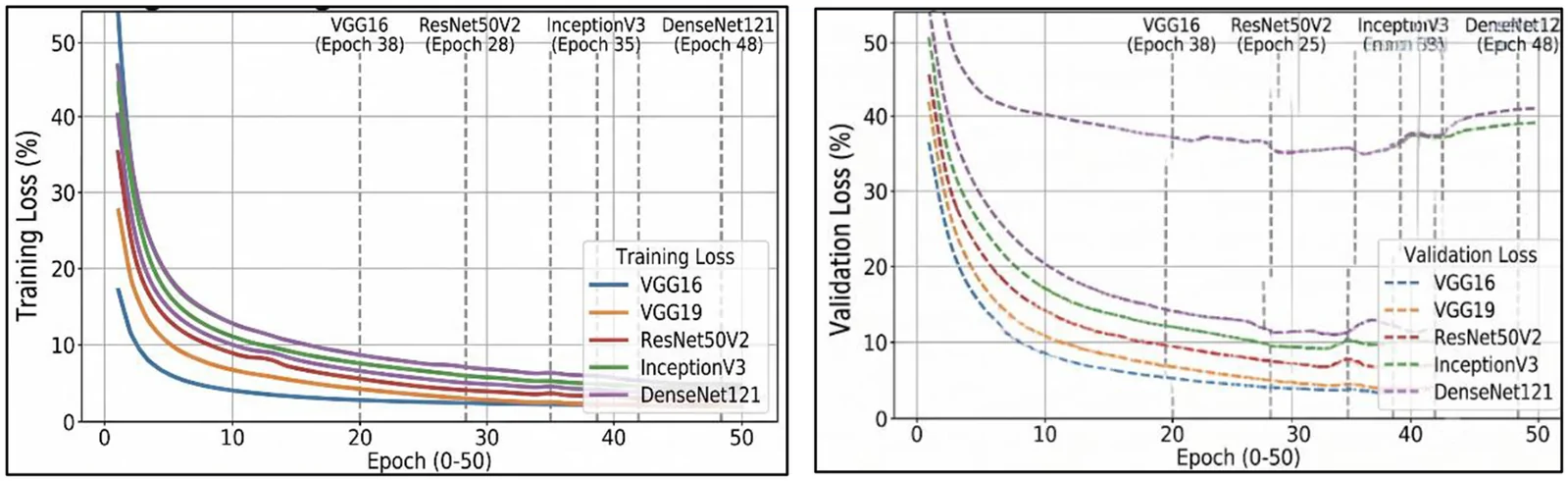
Training and validation loss curves across 50 epochs for all five DCNN architectures, showing convergence behavior and the gap between training and validation loss as an indicator of generalization.

The training and validation accuracy and loss curves for all five DCNN architectures over 50 epochs are presented in Figures 9, 10. All models employed a two-phase training strategy: an initial frozen-base phase followed by partial fine-tuning, indicated by the dashed vertical lines in the figures.

ResNet50V2 and DenseNet121 demonstrated the fastest initial convergence under the frozen-base phase, achieving validation accuracy above 94% within the first five epochs, attributed to their bottleneck and dense-connectivity feature representations. InceptionV3 showed similarly rapid early convergence. VGG16 and VGG19 exhibited more gradual training accuracy trajectories (~88%−89% at end of Phase 1), reflecting the larger fully-connected classification head requiring more iterations to align with the rich frozen convolutional feature representations. Following fine-tuning (Phase 2), all five models showed accelerated improvement in both accuracy and loss, converging to test-set performance levels reported in Section 3.2. The small gap between training and validation curves throughout both phases indicates effective regularization and the absence of significant overfitting across all architectures.

### 3.2 Overall test set performance

**Figure 11:**
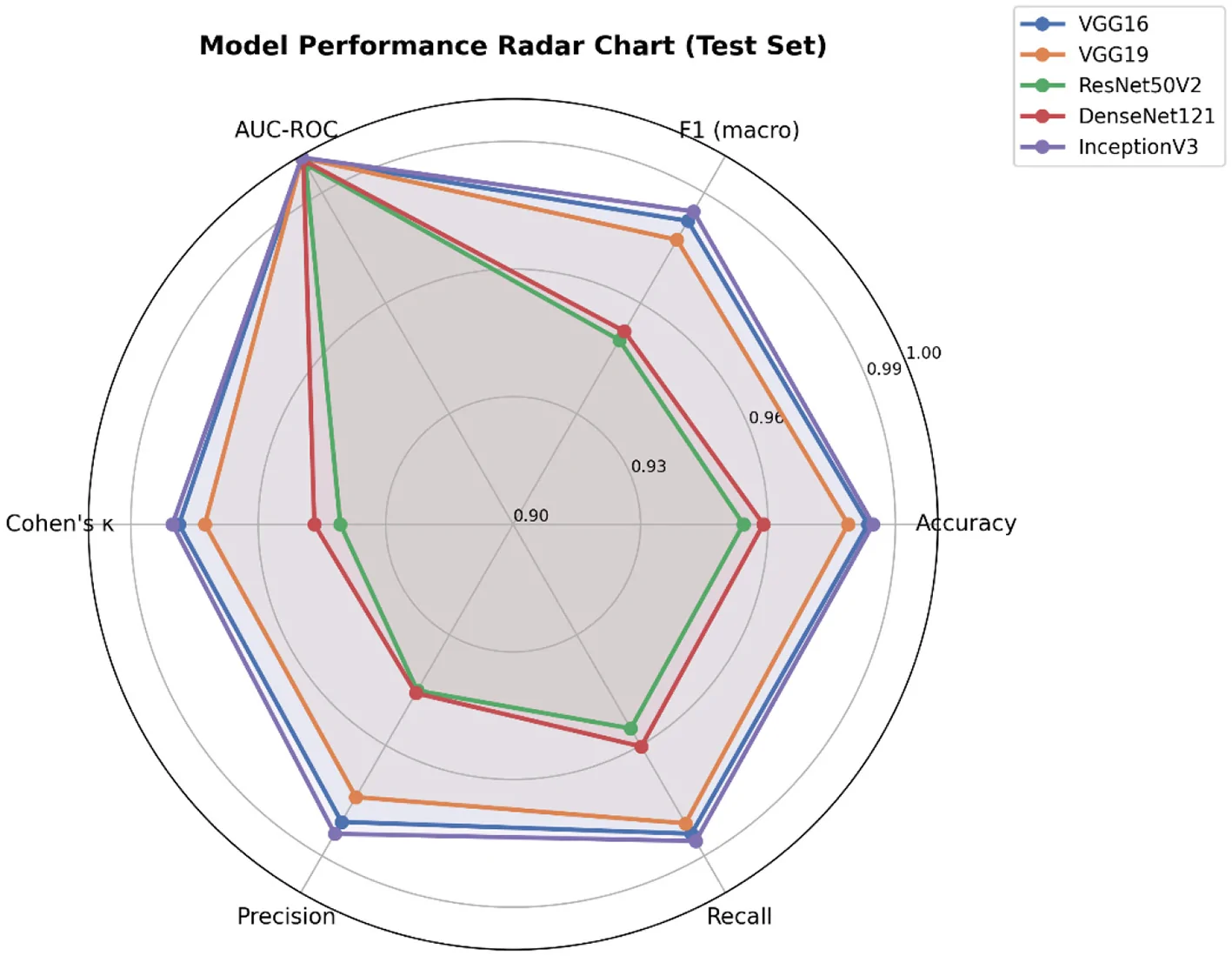
Multi-metric performance radar chart of the evaluated DCNN architectures on the independent test set.

Table 8 presents the complete test-set performance of all five DCNN architectures. InceptionV3 achieved the highest test accuracy (98.47%) and macro-F1 score (98.49%), followed by VGG16 (98.36% accuracy, 98.23% F1) and VGG19 (97.89% accuracy, 97.72% F1). DenseNet121 and ResNet50V2 achieved accuracies of 95.89% and 95.42%, respectively, with macro-F1 scores of 95.24% and 95.00%. AUC-ROC values were consistently high across all five models, ranging from 0.9978 (ResNet50V2) to 0.9995 (InceptionV3), confirming robust class discrimination regardless of decision threshold. Cohen's kappa values mirrored the accuracy ranking, with InceptionV3 (κ = 0.9802) and VGG16 (κ = 0.9787) demonstrating the highest agreement beyond chance, and ResNet50V2 (κ = 0.9407) and DenseNet121 (κ = 0.9468) showing lower but still strong agreement. To visually synthesize these comprehensive evaluation criteria and evaluate the multi-dimensional performance trade-offs of each classifier, a radar chart mapping all six test metrics across the architectures is illustrated in Figure 11.

**Table 8:** Overall test-set performance of five DCNN architectures for mungbean foliar disease classification.

DCNN architecture	Accuracy (%)	Precision (%)	Recall (%)	Macro F1 score (%)	Cohen's κ	AUC-ROC
VGG16	98.36	98.08	98.40	98.23	0.9787	0.9992
VGG19	97.89	97.40	98.12	97.72	0.9726	0.9994
ResNet50V2	95.42	94.51	95.54	95.00	0.9407	0.9978
DenseNet121	95.89	94.58	96.03	95.24	0.9468	0.9988
InceptionV3	98.47	98.40	98.60	98.49	0.9802	0.9995

### 3.3 Class-wise performance analysis of DCNN models

**Figure 12:**
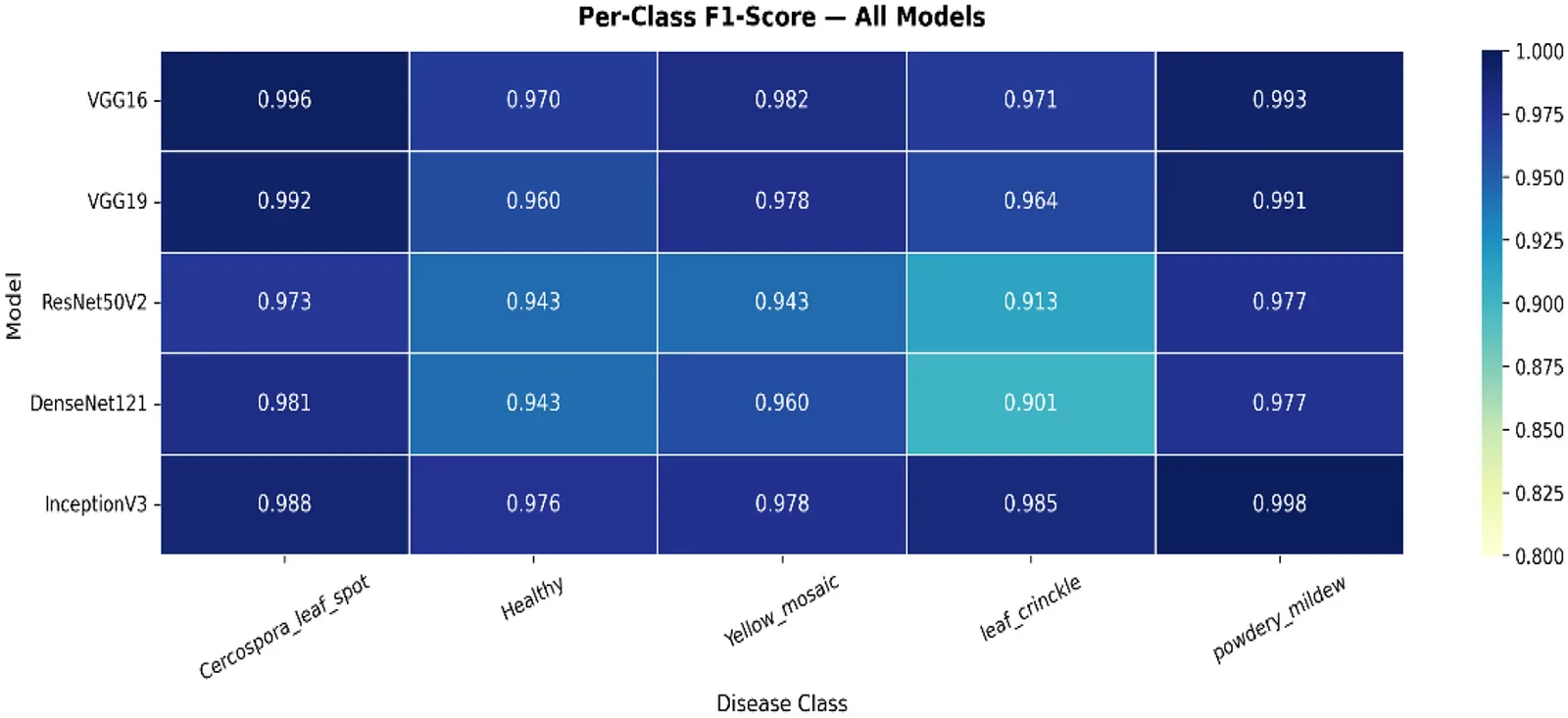
Per-class F1-score heatmap for all five DCNN architectures across five disease categories (healthy, yellow mosaic, powdery mildew, cercospora leaf spot, leaf crinkle).

Per-class F1-scores derived from the test set are visualized as a heatmap in Figure 12. InceptionV3 achieved the highest class-level consistency, with F1-scores of 99.78% for powdery mildew, 98.84% for leaf crinkle, 99.22% for cercospora leaf spot, 97.78% for yellow mosaic, and 97.56% for healthy leaves. VGG16 and VGG19 similarly achieved strong per-class performance, with powdery mildew and cercospora leaf spot consistently the highest-scoring classes. Across all models, powdery mildew was the most distinguishable class, likely attributable to its visually distinctive white powdery surface deposits. Leaf crinkle was the most challenging class overall, reflecting its symptom overlap with early yellow mosaic lesions and variable morphological expression. DenseNet121 and ResNet50V2 exhibited lower F1-scores specifically for leaf crinkle (90.14 and 91.30%, respectively), whereas InceptionV3 achieved 98.84% on this class, suggesting that multi-scale feature extraction is particularly beneficial for diseases with variable lesion patterns.

### 3.4 Statistical significance analysis

**Figure 13:**
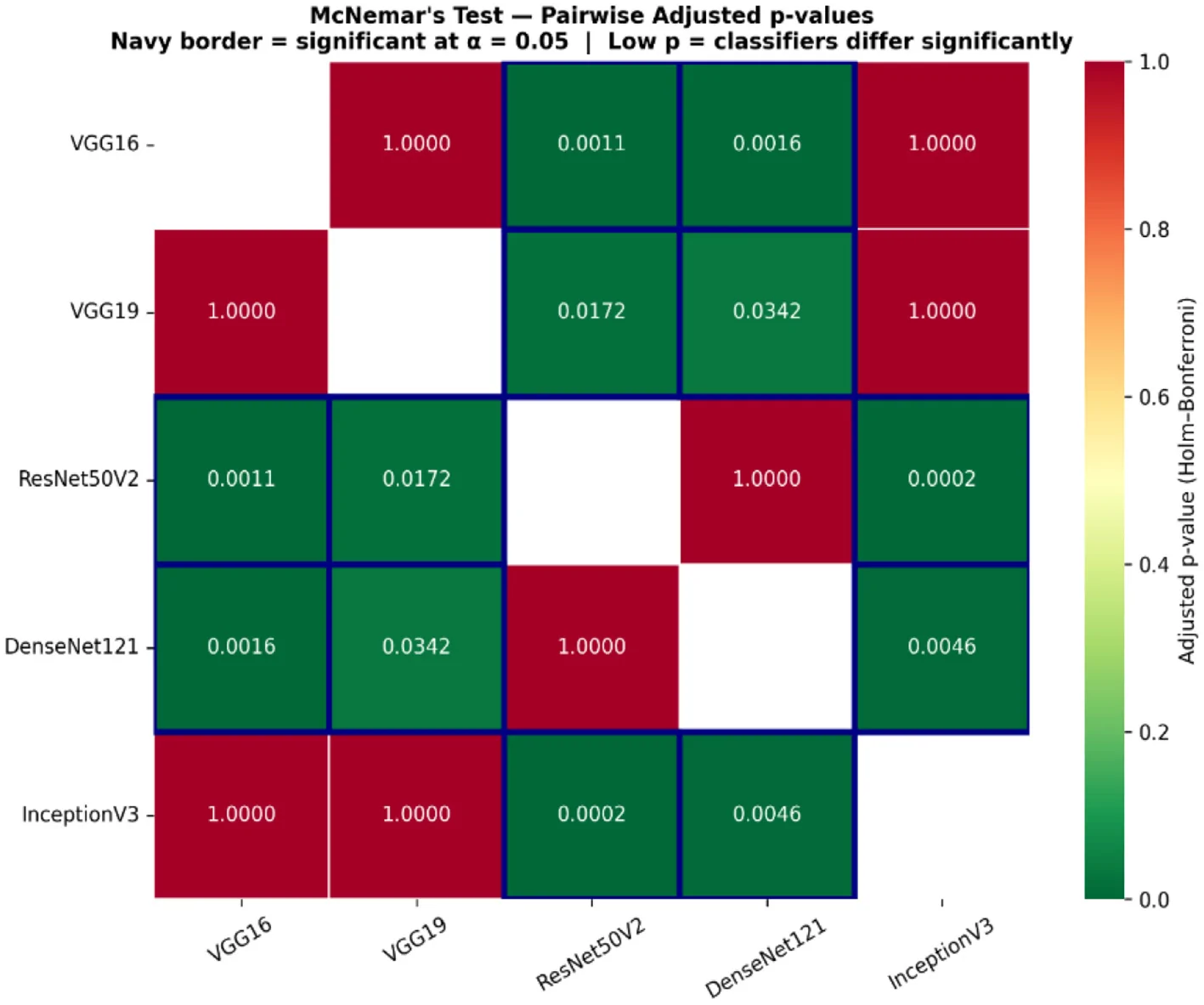
Statistical significance profiling of DCNN classifiers using McNemar's test.

McNemar's test was applied to all 10 pairwise model combinations on the shared test set, with Holm-Bonferroni correction for multiple comparisons (α = 0.05). Results are summarized in Table 9 and visualized as a heatmap in Figure 13. Six of the ten pairwise comparisons reached statistical significance after correction. The three highest-performing models (InceptionV3, VGG16, and VGG19) were not significantly different from each other (p_*a*_d_*j*_ >0.05 for all three pairs), indicating statistically equivalent classification performance on this test set. Similarly, ResNet50V2 and DenseNet121 were not significantly different from each other (*p* = 0.665). However, both InceptionV3 and VGG16 were significantly superior to ResNet50V2 and DenseNet121 (p_*a*_d_*j*_ < 0.05), and InceptionV3 was significantly superior to both ResNet50V2 and DenseNet121. These findings indicate that the performance advantage of InceptionV3, VGG16, and VGG19 over ResNet50V2 and DenseNet121 is statistically robust and not attributable to chance.

**Table 9:** McNemar's test results for pairwise model comparisons (Holm-Bonferroni corrected, α = 0.05).

Model A	Model B	Acc A	Acc B	χ^2^	*p*-adjusted	Significant
VGG16	VGG19	0.9836	0.9789	0.50	1.000	No
VGG16	ResNet50V2	0.9836	0.9542	14.77	0.0011	Yes
VGG16	DenseNet121	0.9836	0.9589	13.79	0.0016	Yes
VGG16	InceptionV3	0.9836	0.9847	0.00	1.000	No
VGG19	ResNet50V2	0.9789	0.9542	8.89	0.0172	Yes
VGG19	DenseNet121	0.9789	0.9589	7.31	0.0342	Yes
VGG19	InceptionV3	0.9789	0.9847	0.64	1.000	No
ResNet50V2	DenseNet121	0.9542	0.9589	0.19	1.000	No
ResNet50V2	InceptionV3	0.9542	0.9847	18.38	0.0002	Yes
DenseNet121	InceptionV3	0.9589	0.9847	11.61	0.0046	Yes

**Figure 14:**
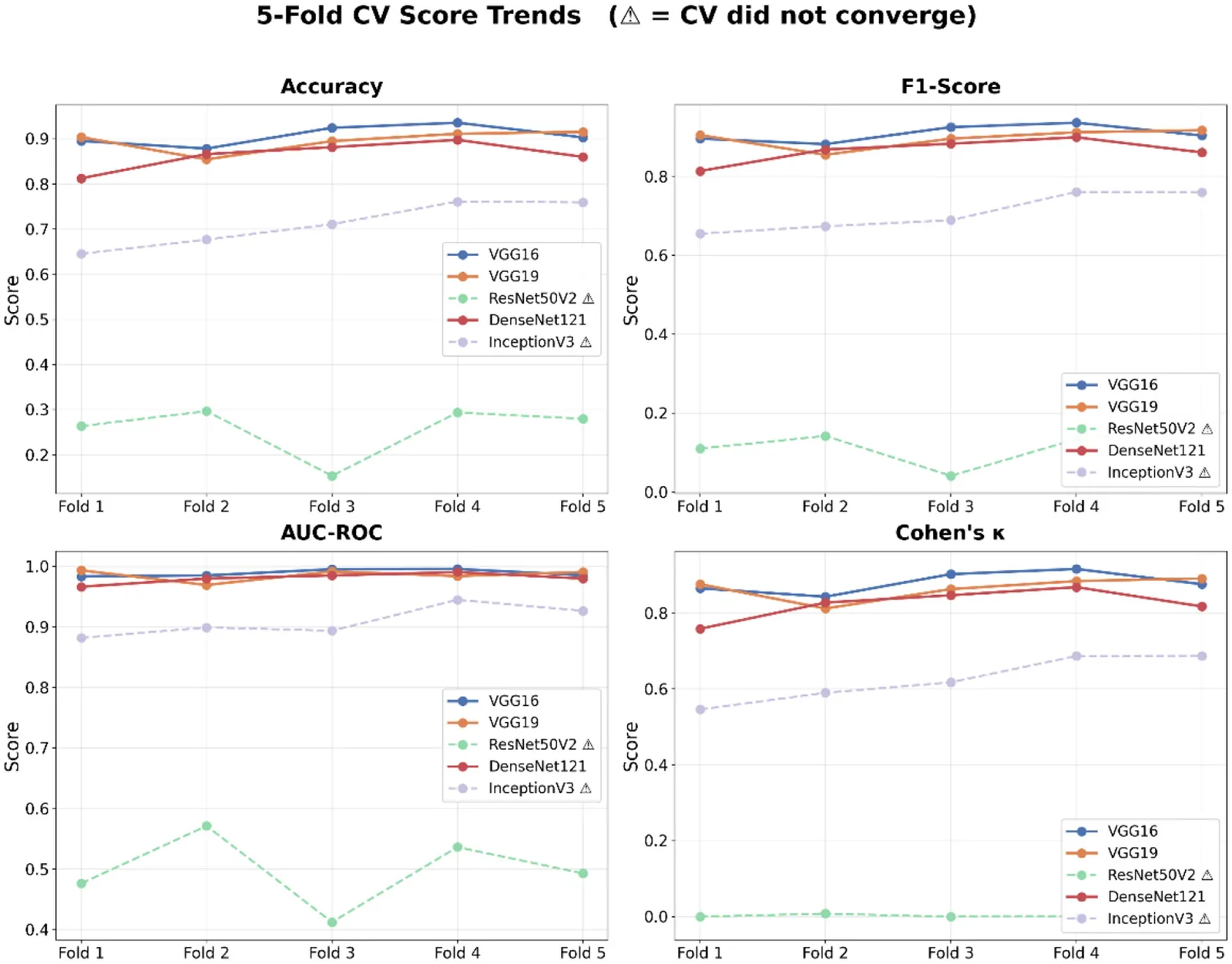
Evaluation of model stability and generalization across five-fold cross-validation.

Five-fold cross-validation was completed for VGG16, VGG19, and DenseNet121, yielding mean CV accuracies of 90.78 ± 2.1%, 89.52 ± 2.1%, and 86.30 ± 2.9%, respectively. CV for ResNet50V2 (CV accuracy: 25.73%) and InceptionV3 (CV accuracy: 71.03%) showed anomalous convergence under the constrained CV training budget and are excluded from CV-based statistical comparisons; their test-set metrics remain valid. Inspection of fold-wise training logs revealed convergence failures in several ResNet50V2 and InceptionV3 folds under the constrained cross-validation training budget, suggesting that these architectures required either larger effective training subsets or extended optimization schedules to achieve stable convergence. This behavior reflects underfitting arising from the fixed, reduced per-fold epoch budget imposed for computational tractability during five-fold cross-validation, rather than an intrinsic limitation of the ResNet50V2 or InceptionV3 architectures. In contrast, the independent held-out test-set evaluation (Section 3.2) used the fully fine-tuned two-phase training protocol described in Section 2.4, and it is this test-set performance—InceptionV3 accuracy of 98.47%—that reflects the true classification capacity of the fully trained model; the CV-fold accuracies for ResNet50V2 and InceptionV3 should therefore not be interpreted as representative of final model performance. Friedman's test on the three convergent models yielded **χ**^**2**^ = 5.20 (*p* = 0.074), indicating no statistically significant difference in CV accuracy among VGG16, VGG19, and DenseNet121. Wilcoxon signed-rank *post-hoc* tests confirmed no significant pairwise differences after Bonferroni correction (Figure 14). The higher test-set accuracy of VGG16 relative to DenseNet121 may reflect beneficial properties of the full training set not fully captured under CV conditions.

### 3.5 Model complexity and computational efficiency

Table 10 presents the complexity and inference characteristics of all five architectures. DenseNet121 is the most parameter-efficient model with only 7.70 M total parameters and 1.72 M trainable parameters, resulting in the smallest model size (29.37 MB). InceptionV3 achieves the highest accuracy at a moderate inference time (78.5 ms per image). VGG16 and VGG19 have larger model sizes (57.65 and 77.91 MB, respectively) but competitive inference times and the advantage of large spatial feature maps for Grad-CAM analysis. ResNet50V2 is the largest model by file size (94.41 MB).

**Table 10:** Model complexity and inference performance.

Model	Total params (M)	Trainable params (M)	Size (MB)	Inference (ms)	FPS	Batch-32 (ms)
VGG16	15.11	13.97	57.65	73.9	13.5	191.7
VGG19	20.42	18.10	77.91	64.2	15.6	199.2
ResNet50V2	24.75	9.06	94.41	64.4	15.5	283.3
DenseNet121	7.70	1.72	29.37	77.3	12.9	704.3
InceptionV3	22.99	14.80	87.69	78.5	12.7	545.2

### 3.6 Confusion matrix analysis and misclassification insights

**Figure 15:**
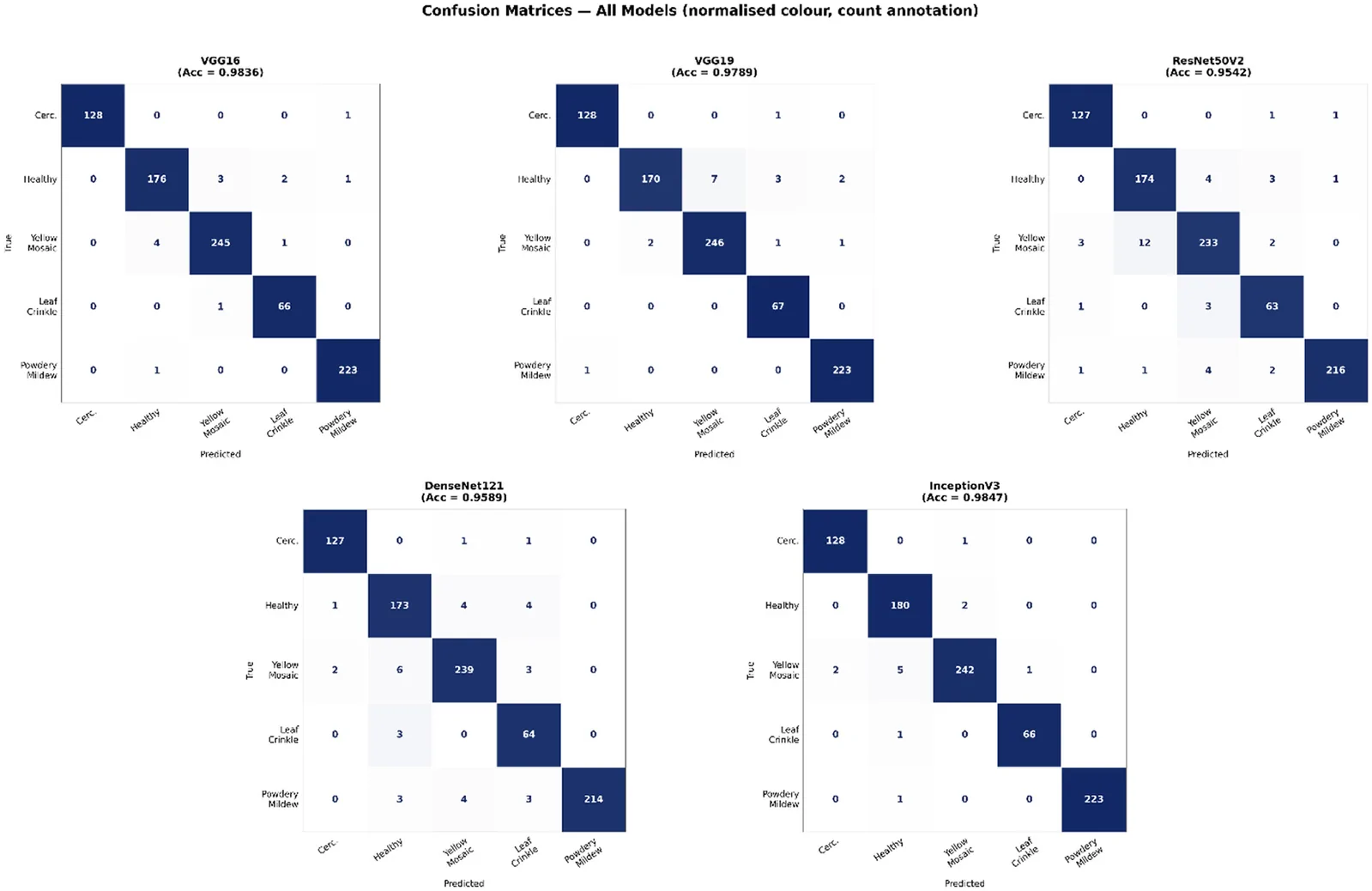
Comparative multi-class confusion matrices for the evaluated deep learning architectures.

Confusion matrices for all five DCNN architectures are presented in Figure 15. InceptionV3 showed the most consistent classification performance, with true-positive rates above 96% across all five disease categories. Only minimal confusion was observed between early yellow mosaic symptoms and healthy leaves, and between leaf crinkle and cercospora leaf spot, in cases of mild or overlapping symptom expression. VGG16 and VGG19 also showed very low misclassification rates, with the majority of errors concentrated in the healthy class. ResNet50V2 showed slightly elevated misclassification for yellow mosaic and powdery mildew, consistent with its lower test accuracy. DenseNet121 exhibited the most notable misclassification pattern for leaf crinkle, consistent with its lowest per-class F1 on leaf crinkle (90.14%). Across all models, misclassifications were distributed across observations rather than concentrated in systematic biases, confirming that all models learned genuine disease-discriminative features.

### 3.7 ROC curve analysis

**Figure 16:**
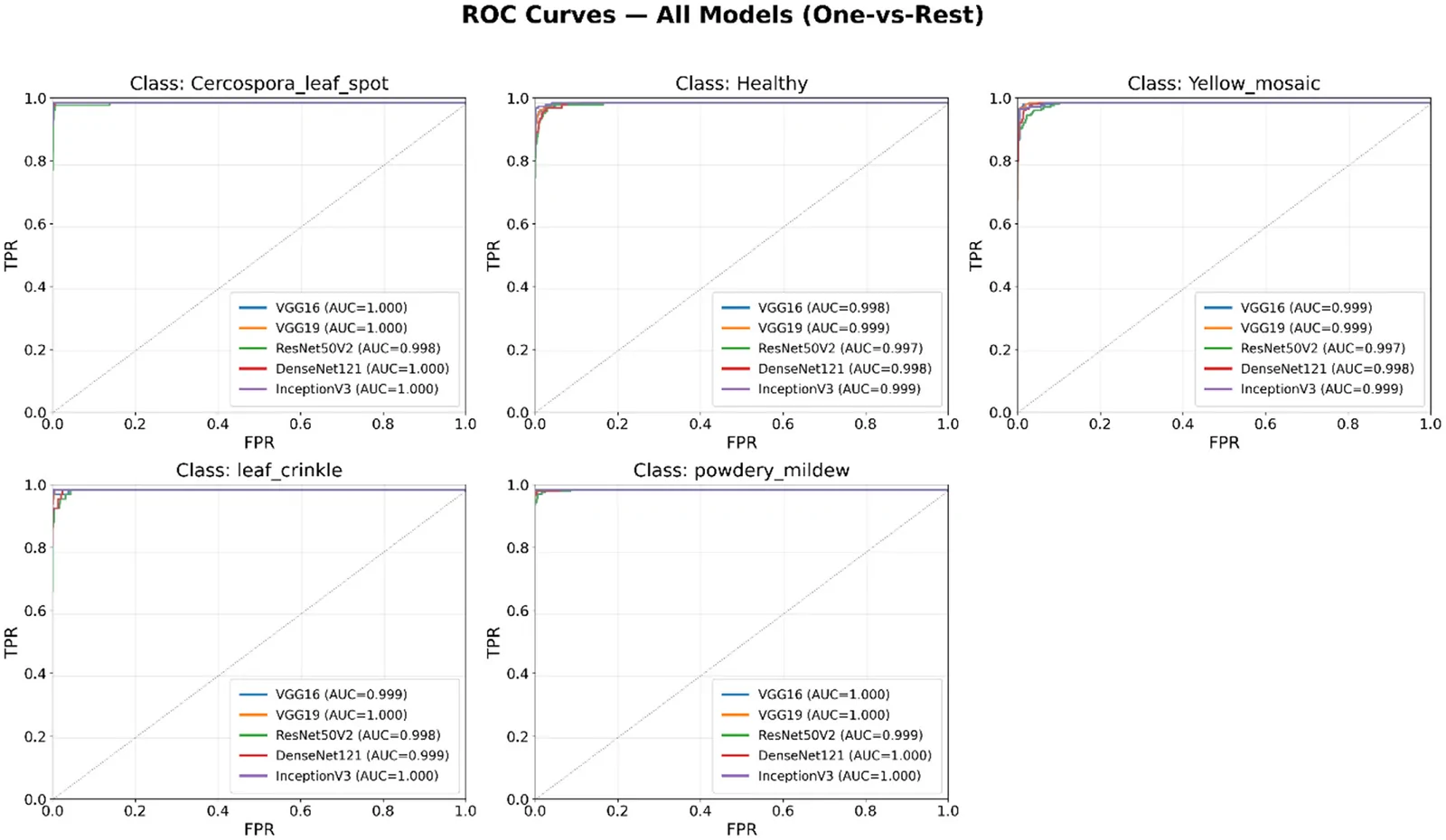
Overlaid ROC curves for all five DCNN architectures across the five disease classes (one-vs.-rest decomposition).

ROC curves for all five architectures across all five disease classes (one-vs.-rest) are presented in Figure 16. All models achieved AUC values above 0.997 across all class-specific ROC curves, with InceptionV3, VGG16, and VGG19 reaching near-perfect AUC = 1.000 for powdery mildew and cercospora leaf spot. The ROC curves for leaf crinkle showed the widest separation between models, with DenseNet121 and ResNet50V2 exhibiting slightly lower AUC values (0.998 and 0.998, respectively) compared to InceptionV3, VGG16, and VGG19 (all 0.999–1.000), consistent with the per-class F1 analysis.

### 3.8 Grad-CAM interpretability analysis

**Figure 17:**
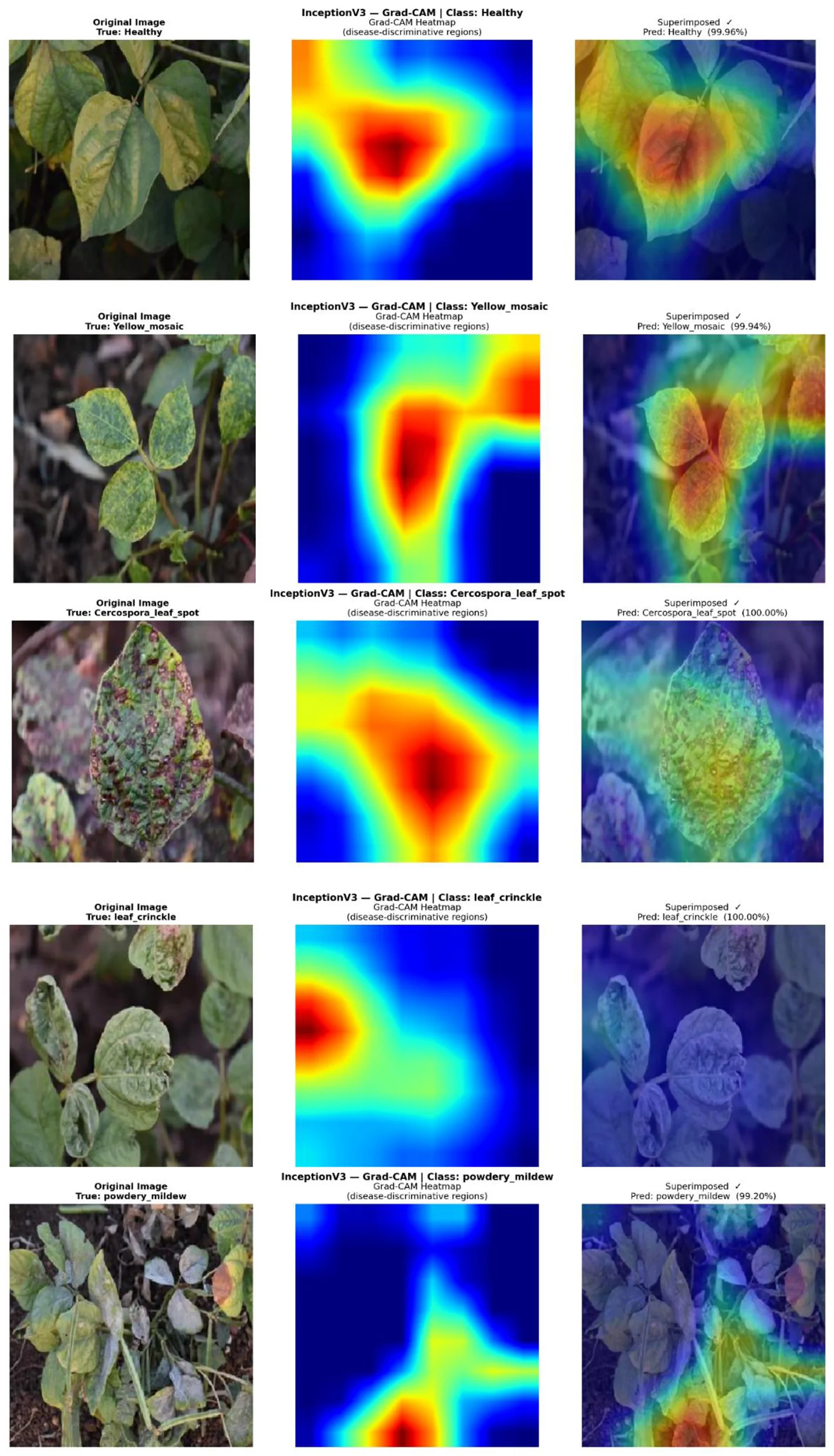
Grad-CAM visualization matrix demonstrating the visual explainability of the top-performing InceptionV3 architecture across all target classes.

To evaluate the visual explainability and biological validity of the network's classification decisions, Gradient-weighted Class Activation Mapping (Grad-CAM) was applied to map layer-specific heatmaps and activation regions across the five disease classes. While all five architectures demonstrated similar target-localization properties during evaluation, InceptionV3 was selected as the representative architecture for visual presentation due to its superior overall test-set accuracy. As illustrated in Figure 17, the comparative sub-figures display the original field images alongside the localized layer-specific heatmaps and superimposed activation maps generated by the network. The consolidated results demonstrate that the model consistently directed its attention toward biologically meaningful, diagnostically relevant foliar regions rather than non-diagnostic background elements, soil, or field artifacts.

For powdery mildew, model activations concentrated precisely on the regions of visible white powdery fungal deposits on the leaf surface, yielding high prediction confidence scores. For leaf crinkle, the network focused heavily on the distorted and crinkled laminar regions along the leaf veins, aligning with the virus-induced vein distortion typical of this disease. In the case of yellow mosaic, the heatmaps accurately highlighted the interveinal chlorotic patches and mosaic discoloration areas. For cercospora leaf spot, model gradients concentrated tightly along the circular lesion boundaries with darker necrotic centers. Conversely, when processing healthy leaves, the architecture displayed a broad, diffuse activation distribution across the entire lamina without concentration on any specific region, confirming the absence of localized, disease-specific patterns.

The strong biological correspondence between the localized Grad-CAM hotspots and the known macroscopic diagnostic features of each condition confirms that the high metric performance achieved stems from genuine feature learning rather than spurious background correlations or lighting artifacts. These interpretability results confirm the robustness of the trained classification pipeline, validate its reliability for practical field use, and substantially improve the interpretability of model predictions.

## 4 Discussion

This study provides a statistically robust, multi-perspective benchmarking framework for the automated identification of major foliar diseases in mungbean using in-field leaf imagery. Unlike traditional deep learning frameworks in agricultural computer vision that rely solely on empirical point-estimates of accuracy, this workflow integrates pairwise hypothesis testing via McNemar's test with Holm-Bonferroni corrections alongside extended metrics such as Cohen's kappa and macro-standardized F1-scores. This mathematical framework provides stronger evidence that the observed performance differences are unlikely to be due to random sampling variation in feature extraction capability rather than random sampling artifacts, addressing a critical gap noted in recent crop phenotyping literature (Richter et al., 2025).

Among the evaluated DCNNs, InceptionV3 emerged as the best-performing classifier, securing a peak test accuracy of 98.47%, a macro-F1 score of 98.49%, and an exceptionally high agreement beyond chance. This superior classification performance is fundamentally tied to its architectural layout. The parallel convolutional blocks within individual Inception modules extract features at variable receptive fields simultaneously. These observations are consistent with earlier findings by Dutta et al. (2024) and Kaur et al. (2024), who also reported high effectiveness of InceptionV3 architectures for classification of different plant diseases.

This multi-scale representational capacity proved uniquely advantageous for complex and morphologically variable symptoms. For instance, InceptionV3 achieved an F1-score of 98.84% on the highly challenging “leaf crinkle” class. Leaf crinkle virus infections induce a compound syndrome characterized by uneven laminar distortion, puckering and curling features that vary dramatically depending on the age of the leaflet and canopy lighting. By evaluating structural features across multiple spatial dimensions, InceptionV3 avoids the spatial resolution bottlenecks common to rigid, single-stream architectures, echoing findings by Mathivanan et al. (2026) on multi-scale agricultural disease profiling.

Interestingly, VGG16 and VGG19 demonstrated competitive statistical equivalence with InceptionV3, yielding high test accuracies of 98.36% and 97.89%, respectively. The trends observed in this study support the conclusions of (Tyagi and Vats, 2024; Reddy and Rajasekar, 2024), who highlighted the strength of VGG16 and VGG19 models in fine-grained disease identification. Pairwise McNemar analysis confirmed that the performance variances among InceptionV3, VGG16 and VGG19 were statistically negligible (Mahmud et al., 2025). While older literature often reports a significant performance drop for VGG architectures when compared to deep residual networks, our results demonstrate that utilizing an initially frozen feature-extraction base followed by selective fine-tuning preserves much of the generalized ImageNet representation while enabling adaptation to disease-specific features. This configuration successfully prevents catastrophic forgetting during the optimization of the classification head, rendering the VGG family highly stable for this specific five-class agricultural task (Das, 2023).

In contrast, DenseNet121 and ResNet50V2 underperformed relative to the top-tier models, achieving significantly lower test accuracies of 95.89% and 95.42%, respectively. The subdued performance of DenseNet121, despite its advanced dense connectivity loops, highlights a unique constraint of the frozen-base transfer learning paradigm. Although DenseNet121 benefits from efficient feature reuse, the limited fine-tuning strategy may have constrained its ability to fully adapt its learned representations to the mungbean disease domain. This limitation is supported by the pronounced discrepancy between its five-fold cross-validation accuracy (86.30%) and its final standalone test-set accuracy (95.89%), pointing to training instabilities under constrained cross-validation budgets (Gomez-Flores et al., 2022).

Nevertheless, from an edge-deployment perspective, DenseNet121 presents a highly optimized architectural footprint. It possesses the lowest memory requirement (29.37 MB) and a highly streamlined total parameter footprint (7.70 M), positioning it as an ideal candidate for integration into low-power mobile applications or remote edge-computing hardware, as corroboratively argued by Hoang et al. (2026). Similarly, the slightly lower accuracy of ResNet50V2 can be attributed to its optimization requirements. Possessing 9.06 M trainable parameters across its classification and residual adaptation boundaries, this architecture exhibited unstable performance across cross-validation folds, suggesting that the architecture may require larger training subsets or alternative optimization settings for reliable convergence (Werku et al., 2024).

A major strength of this study lies in verifying these decision boundaries learned by the model through Gradient-weighted Class Activation Mapping (Grad-CAM). Interpretability profiling of the top-performing InceptionV3 model confirmed a high biological correspondence between its internal focal points and actual macroscopic diagnostic markers (Karim et al., 2024). The network's gradients concentrated strictly on the visible white powdery fungal spots for Powdery Mildew, followed the distorted, crinkled margins along the primary veins for Leaf Crinkle and isolated the circular necrotic boundaries characteristic of Cercospora spots. Crucially, when processing healthy canopies, the network maintained a diffuse, uniform activation across the entire lamina. This lack of localized focusing provides evidence that the pipeline's high classification accuracy is driven by genuine, biologically valid phytopathological features rather than superficial background correlations, soil artifacts, or camera exposure variations.

The present study has several notable strengths. The use of a large field-acquired dataset, leakage-free group-based dataset splitting, and post-split data augmentation enhanced the reliability of model evaluation. Furthermore, the integration of comprehensive performance metrics, statistical validation using McNemar's test with Holm-Bonferroni correction, and Grad-CAM interpretability provided a robust and biologically meaningful assessment of the proposed deep learning framework.

Despite these strong results, several limitations of the present study should be acknowledged. The dataset was collected from a single geographical region and growing environment, which may not fully represent the diversity of disease manifestations encountered across different agro-climatic zones. Although transfer learning with an initially frozen convolutional base followed by partial fine-tuning produced strong results, alternative fine-tuning strategies may further improve feature adaptation and classification performance. In addition, the study focused on image-based disease recognition and did not incorporate complementary sources of information, such as environmental, spectral, or temporal data, that may further enhance diagnostic accuracy.

Building on these findings, future research should focus on collecting larger multi-location and multi-season datasets to improve model generalizability and robustness. The integration of lightweight deep learning architectures suitable for mobile and edge-device deployment should also be explored to facilitate real-time field diagnosis. Furthermore, combining RGB imagery with hyperspectral, multispectral, or environmental sensor data may provide richer representations for disease detection. Additional work on explainable artificial intelligence techniques and automated disease monitoring platforms, including drone- and smartphone-based systems, could further support precision agriculture applications.

## 5 Conclusion

This study presented a comprehensive deep learning framework for automated classification of major foliar diseases in mungbean using field-acquired image data. Five state-of-the-art DCNN architectures, namely VGG16, VGG19, ResNet50V2, DenseNet121, and InceptionV3, were systematically evaluated under identical transfer learning conditions. The proposed framework incorporated rigorous dataset preparation, post-split data augmentation, class-weighted learning, and extensive performance evaluation using multiple statistical and machine learning metrics. The results demonstrated that all evaluated architectures were capable of accurately discriminating among healthy leaves, yellow mosaic disease, powdery mildew, leaf crinkle, and cercospora leaf spot under natural field conditions.

The major contributions of this work include the development of a reproducible benchmarking framework for mungbean foliar disease classification using a large-scale field-based dataset and the incorporation of comprehensive evaluation measures beyond conventional accuracy. In addition to precision, recall, and F1-score, the study employed AUC-ROC analysis, Cohen's kappa coefficient, model complexity assessment, McNemar's pairwise significance testing, and Grad-CAM visualizations to provide a more rigorous understanding of model behavior. The results revealed that InceptionV3 achieved the highest test accuracy (98.47%), macro-F1 (98.49%), AUC-ROC (0.9995), and Cohen's kappa (0.9802), attributable to its multi-scale feature extraction capability through parallel Inception modules. VGG16 and VGG19 performed statistically equivalently to InceptionV3, while ResNet50V2 and DenseNet121 were significantly lower-performing on this dataset under the adopted transfer-learning and fine-tuning protocol. DenseNet121 distinguished itself as the most parameter-efficient architecture, offering the best accuracy-per-parameter trade-off and representing a promising candidate for future edge deployment. All models achieved AUC-ROC values above 0.997 and Cohen's kappa above 0.94, confirming strong class discrimination and agreement well beyond chance.

The advantages of the proposed framework lie in its ability to operate under realistic field conditions characterized by diverse backgrounds, lighting variations, symptom severities, and plant growth stages. The use of statistically validated comparisons enabled identification of genuine performance differences among architectures, while the field-acquired dataset improves the practical relevance of the findings for real-world disease monitoring applications. The results provide valuable insights into the suitability of different DCNN architectures for mungbean disease classification and offer a useful foundation for future deep learning research in mungbean and other pulse crops.

In conclusion, this study demonstrates the effectiveness of deep learning-based approaches for accurate and reliable mungbean foliar disease classification under natural field conditions. By providing a statistically validated comparison of multiple DCNN architectures, the findings highlight the feasibility of deploying AI-based tools for crop health monitoring and support the development of sustainable, data-driven disease management practices in precision agriculture.

## Data Availability

The raw data supporting the conclusions of this article will be made available by the authors, without undue reservation.

## References

[B1] Arnal Barbedo J. G. (2013). Digital image processing techniques for detecting, quantifying and classifying plant diseases. SpringerPlus 2, 660. doi: 10.1186/2193-1801-2-660 24349961 PMC3863396

[B2] Brahimi M. Arsenovic M. Laraba S. Sladojevic S. Boukhalfa K. Moussaoui A. (2018). “Deep learning for plant diseases: detection and saliency map visualisation,” in: *Human and Machine Learning*, 93–117.

[B3] Chauhan A. Parihar A. Singh S. (2025). From leaves to lab: innovative methods in plant disease diagnosis. Int. J. Eng. Computer Sci. 7, 219–226. doi: 10.33545/26633582.2025.v7.i1c.184

[B4] Das P. K. (2023). Leaf disease classification in bell pepper plant using VGGNet. J. Innov. Image Proc. 5, 36–46. doi: 10.36548/jiip.2023.1.003

[B5] Deb N. Rahman T. (2025). An efficient VGG16-based deep learning model for automated potato pest detection. Smart Agricultural Technol. 12:101409. doi: 10.1016/j.atech.2025.101409

[B6] Devi P. I. Manjula M. Bhavani R. V. (2022). Agrochemicals, environment, and human health. Annual Rev. Environ. Res. 47, 399–421. doi: 10.1146/annurev-environ-120920-111015

[B7] Dolatabadian A. Neik T. X. Danilevicz M. F. Upadhyaya S. R. Batley J. Edwards D. (2025). Image-based crop disease detection using machine learning. Plant Pathol. 74, 18–38. doi: 10.1111/ppa.14006

[B8] Dorling D. (2021). “World population prospects at the UN: our numbers are not our problem?,” in: *The Struggle for Social Sustainability*. (Policy Press) 129–154.

[B9] Dubey S. C. Singh B. (2013). Integrated management of major diseases of mungbean by seed treatment and foliar application of insecticide, fungicides and bioagent. Crop Protection 47, 55–60. doi: 10.1016/j.cropro.2012.12.025

[B10] Dutta M. Gupta D. Gulzar Y. Mir M. S. Onn C. W. Soomro A. B. (2024). Leveraging inception V3 for precise early and late blight disease classification in potato crops. Traitement Du Signal 41, 705–715. doi: 10.18280/ts.410213

[B11] Ferentinos K. P. (2018). Deep learning models for plant disease detection and diagnosis. Comput. Electr. Agricul. 145, 311–318. doi: 10.1016/j.compag.2018.01.009

[B12] Gomez-Flores W. Garza-Saldana J. J. Varela-Fuentes S. E. (2022). A huanglongbing detection method for orange trees based on deep neural networks and transfer learning. IEEE Access 10, 116686–116696. doi: 10.1109/ACCESS.2022.3219481

[B13] He K. Zhang X. Ren S. Sun J. (2016). Deep residual learning for image recognition. 2016 IEEE conference on computer vision and pattern recognition (CVPR) 770–778. doi: 10.1109/CVPR.2016.90

[B14] Hoang T.-M. Bui V.-H. Nguyen V.-S. Doan D.-T. Dang H.-A. Pham A.-T. (2026). A comprehensive evaluation of lightweight deep learning models for tomato disease classification on edge computing environments. Sci. Rep. 16:12320. doi: 10.1038/s41598-026-42439-6 41786837 PMC13079885

[B15] Huang G. Liu Z. Van Der Maaten L. Weinberger K. Q. (2017). Densely connected convolutional networks. 2017 IEEE conference on computer vision and pattern recognition (CVPR) 2261–2269. doi: 10.1109/CVPR.2017.243

[B16] Huang X. Lin D. Mao X. Zhao Y. (2023). Multi-source data fusion for estimating maize leaf area index over the whole growing season under different mulching and irrigation conditions. Field Crops Res. 303:109111. doi: 10.1016/j.fcr.2023.109111

[B17] Huppertz M. Manasa S. L Kachhap D. Dalai A. Yadav N. Baby D. . (2023). Exploring the potential of mung bean: sustainability and genomics in a changing world. *J. Agricul. Food Res*. 14:100786. doi: 10.1016/j.jafr.2023.100786

[B18] Kamilaris A. Prenafeta-Boldú F. X. (2018). Deep learning in agriculture: a survey. Comput. Electr. Agricul. 147, 70–90. doi: 10.1016/j.compag.2018.02.016

[B19] Kamireddy R. Lakra N. Ahlawat Y. Sundaramoorthy M. Menon S. V. Manorama K. . (2025). Integrated morphological, biochemical and molecular screening of mungbean (*Vigna radiata* L.) genotypes for mungbean yellow mosaic virus (MYMV) resistance. Plant Physiology and Biochemistry 229, 110596. doi: 10.1016/j.plaphy.2025.110596 41265022

[B20] Kanade A. K. Potdar M. P. Balol G. Rathod N. P. Huligol S. N. Channakeshava R. . (2025). Artificial Intelligence based Precise Disease Detection in Soybean using Real Time Object Detectors. LEGUME RESEARCH - AN INTERNATIONAL JOURNAL, (Of). doi: 10.18805/LR-5431

[B21] Karim M.d,. J Goni Md,. O. F. Nahiduzzaman M.d. Ahsan M. Haider J. Kowalski M. (2024). Enhancing agriculture through real-time grape leaf disease classification via an edge device with a lightweight CNN architecture and Grad-CAM. Scientific Reports 14, 16022. doi: 10.1038/s41598-024-66989-9 38992069 PMC11239930

[B22] Kaur A. Kukreja V. Kumar M. Choudhary A. Sharma R. (2024). An automatic fine-tuned inceptionv3 model for groundnut leaf disease multiclass classification. 2024 5th international conference for emerging technology (INCET), 1–6. doi: 10.1109/INCET61516.2024.10593146

[B23] Kheir A. M. S. Koubaa A. Kolluru V. Mungara S. Feike T. (2025). Smart plant disease diagnosis using multiple deep learning and web application integration. J. Agricul. Food Res. 21:101948. doi: 10.1016/j.jafr.2025.101948

[B24] Kopittke P. M. Menzies N. W. Wang P. McKenna B. A. Lombi E. (2019). Soil and the intensification of agriculture for global food security. Environ. Int. 132:105078. doi: 10.1016/j.envint.2019.105078 31400601

[B25] Liakos K. Busato P. Moshou D. Pearson S. Bochtis D. (2018). Machine learning in agriculture: a review. Sensors 18:2674. doi: 10.3390/s18082674 30110960 PMC6111295

[B26] Mahmud T. Akter M.st,. S Akter T. Shahadat Hossain M. Andersson K. (2025). Exploring deep learning and explainable AI for precise rice leaf disease detection and classification. IEEE Access 13, 162821–162844. doi: 10.1109/ACCESS.2025.3609671

[B27] Mallick M. T. Banerjee S. Thakur N. Saha H. N. Chakrabarti A. (2025). Evaluation of state-of-the-art deep learning techniques for plant disease and pest detection. Comput. Mat. Continua 85, 121–180. doi: 10.32604/cmc.2025.065250

[B28] Mathivanan B. Santhuja P. Rubinabegam M. Muthulekshmi Balasubramani, S. Dhanush S. (2026). Detecting sugarcane leaf diseases using inceptionv3 convolutional neural networks. 2026 international conference on AI-driven smart systems and ubiquitous computing (ICAUC), 1439–1444. doi: 10.1109/ICAUC68182.2026.11441235

[B29] McKenzie F. C. Williams J. (2015). Sustainable food production: constraints, challenges and choices by 2050. Food Security 7, 221–233. doi: 10.1007/s12571-015-0441-1

[B30] Mohanty S. P. Hughes D. P. Salathé M. (2016). Using deep learning for image-based plant disease detection. Front. Plant Sci. 7:1419. doi: 10.3389/fpls.2016.01419 27713752 PMC5032846

[B31] Pandey A. K. Burlakoti R. R. Kenyon L. Nair R. M. (2018). Perspectives and challenges for sustainable management of fungal diseases of mungbean [*Vigna radiata* (L.) R. Wilczek var. radiata]: a review. Front. Environ. Sci. 6:53. doi: 10.3389/fenvs.2018.00053

[B32] Parasappa Saable S. Revanappa B. Pavan S Manu B. Channamma K. Ramakrishnan N. . (2024). Response of mungbean genotypes for resistance against mungbean yellow mosaic virus. J. Food Legumes 37, 90–94. doi: 10.59797/jfl.v37.i1.181

[B33] Powers D. M. W. (2020). Evaluation: from precision, recall and F-measure to ROC, informedness, markedness and correlation.

[B34] Reddy N. K. Rajasekar M. (2024). “Increasing F1 score with VGG16 during plant disease classification over VGG19,” in AIP Conference Proceedings, Vol. 3193, 020191. doi: 10.1063/5.0238123

[B35] Richter D. J. Ilias Bappi M. Sanjay Kolekar S. Kim K. (2025). A systematic review of the current state of transfer learning accelerated CNN-based plant leaf disease classification. IEEE Access 13, 116262–116303. doi: 10.1109/ACCESS.2025.3584404

[B36] Rumpf T. Mahlein A.-K. Steiner U. Oerke E.-C. Dehne H.-W. Plümer L. (2010). Early detection and classification of plant diseases with support vector machines based on hyperspectral reflectance. Comput. Electronics in Agricul. 74, 91–99. doi: 10.1016/j.compag.2010.06.009

[B37] Shorten C. Khoshgoftaar T. M. (2019). A survey on image data augmentation for deep learning. J. Big Data 6:60. doi: 10.1186/s40537-019-0197-0 34306963 PMC8287113

[B38] Simonyan K. Zisserman A. (2015). Very deep convolutional networks for large-scale image Recognition, *in ICLR*. arXiv:1409.1556 .

[B39] Szegedy C. Vanhoucke V. Ioffe S. Shlens J. Wojna Z. (2016). Rethinking the inception architecture for computer vision. 2016 IEEE conference on computer vision and pattern recognition (CVPR) 2818–2826. doi: 10.1109/CVPR.2016.308

[B40] Too E. C. Yujian L. Njuki S. Yingchun L. (2019). A comparative study of fine-tuning deep learning models for plant disease identification. Comput. Electronics Agricult. 161, 272–279. doi: 10.1016/j.compag.2018.03.032

[B41] Tugrul B. Elfatimi E. Eryigit R. (2022). Convolutional neural networks in detection of plant leaf diseases: a review. Agriculture 12:1192. doi: 10.3390/agriculture12081192

[B42] Tyagi K. Vats S. (2024). Implementing inception v3, VGG-16 and VGG-19 architectures of CNN for medicinal plant leaves identification and disease detection. J. Electr. Syst. 2380–2388. doi: 10.52783/jes.3989

[B43] Upadhyay A. Chandel N. S. Singh K. P. Chakraborty S. K. Nandede B. M. Kumar M. . (2025). Deep learning and computer vision in plant disease detection: a comprehensive review of techniques, models, and trends in precision agriculture. Artif. Intel. Rev. 58:92. doi: 10.1007/s10462-024-11100-x

[B44] Vinod C. S. S. Anand H. S. Albaaji G. F. (2024). Precision farming for sustainability: an agricultural intelligence model. Comput. Electro. Agricul. 226:109386. doi: 10.1016/j.compag.2024.109386

[B45] Werku K. N. Kassa I. G. Desta A. B. Ayal D. M. Bimrew M. A. Ayele M. K. (2024). Classification of angular leaf spot, bean rust diseases in haricot bean leaves using CNN and transfer learning. 2024 international conference on information and communication technology for development for Africa (ICT4DA) 62–67. doi: 10.1109/ICT4DA62874.2024.10777237

